# Synthesis of Tris(Dimethylamino)Phosphine‐Based InP Quantum Dots and Their Application in Light‐Emitting Diodes: Progress and Perspectives

**DOI:** 10.1002/exp2.70182

**Published:** 2026-05-25

**Authors:** Zifeng Zhang, Jilin Deng, Qiulei Xu, Zhenghui Wu, Yanbing Lv, Baocheng Yang, Haiyang Li, Fei Chen, Huaibin Shen

**Affiliations:** ^1^ Henan Provincial Key Laboratory of Nanocomposites and Applications Institute of Nano‐Structured Functional Materials, Huanghe Science and Technology College Zhengzhou China; ^2^ Key Lab for Special Functional Materials of Ministry of Education, School of Nanoscience and Materials Engineering Henan University Kaifeng China; ^3^ Sungkyunkwan University (SKKU) Suwon Republic of Korea

**Keywords:** (DMA)_3_P, InP quantum dot, quantum dot light‐emitting diodes, progress, perspective

## Abstract

Indium phosphide (InP)‐based quantum dots (QDs) have emerged as promising cadmium‐free alternatives for next‐generation optoelectronic applications, particularly in quantum dot light‐emitting diodes (QLEDs). Tris(dimethylamino)phosphine ((DMA)_3_P) has gained attention as a low‐toxicity alternative to conventional precursors like tris(trimethylsilyl)phosphine ((TMS)_3_P) or toxic phosphine gas (PH_3_) in the synthesis of InP QDs. However, InP core/shell QDs synthesized using (DMA)_3_P and their corresponding QLEDs currently exhibit inferior optical and electronic performance compared to their (TMS)_3_P‐based counterparts. This review provides a comprehensive analysis of the molecular structures and distinct reaction mechanisms of (TMS)_3_P and (DMA)_3_P during InP core nucleation. Then, we systematically address the key challenges in optimizing (DMA)_3_P‐derived InP QDs, including defect state passivation and carrier confinement, and summarize effective improvement strategies encompassing core modulation, core/shell structure design, and surface ligand engineering. Furthermore, we discuss critical issues in integrating these QDs into QLEDs, focusing on charge transport engineering and suppression of charge leakage. Finally, we outline the remaining challenges and prospects for advancing InP‐based QLEDs in displays and solid‐state lighting.

## Graphical abstract

A lot of subtle but key tricks in the synthesis of (DMA)_3_P‐based InP QDs and device mechanisms were not explicitly explained in the published works.

This review firstly provides a comprehensive analysis of the molecular structures and distinct reaction mechanisms of (TMS)_3_P and (DMA)_3_P during InP core nucleation. Then, this review systematically addresses the key challenges and proposes effective improvement strategies in optimizing (DMA)_3_P‐derived InP QDs and corresponding QLEDs.

This review, if published, would greatly help those scholars who want to quickly get on track with the research about (DMA)_3_P‐based InP QDs and QLEDs.

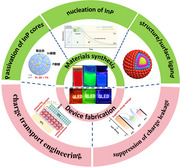

## 1 Introduction

Emerging display technologies are characterized by the enhancement in picture quality, form flexibility, and energy efficiency. Notably, quantum dot light‐emitting diodes (QLEDs), which employ colloidal quantum dots (QDs) as the emitting material, attain higher brightness and a wider color gamut. Furthermore, it is not susceptible to screen burn‐in, a limitation associated with organic light‐emitting diodes (OLEDs) [1]. As a result, QD‐based electroluminescent devices are expected to become the next‐generation mainstream light‐emitting display technology, making them a hotly contested field worldwide [2, 3, 4, 5, 6, 7, 8, 9]. Since the first report of QLEDs in 1994 [2], some key performance metrics of the three primary color QLEDs have approached or met the requirements for industrial applications [10, 11, 12, 13, 14, 15, 16, 17, 18]. However, current high‐performance QLEDs typically use Cd/Pb‐based QDs as the light‐emitting materials, and the inherent toxicity of Cd/Pb elements imposes strict limitations on their practical applications. Therefore, extensive research has been devoted to developing greener alternatives, including I‐III‐VI QDs, III‐V QDs, and carbon QDs. Nevertheless, I‐III‐VI QDs exhibit several limitations, such as intricate synthesis procedures, sensitivity to multiple reaction parameters, and inadequate stability [19, 20]. Meanwhile, carbon QDs still face challenges in achieving comparable color purity and a broad luminescence tuning range [21, 22]. Among them, indium phosphide (InP)‐based QDs have risen to prominence as the most technologically viable and environmentally benign alternative. They demonstrate exceptional photophysical properties, including: (1) full‐spectrum tunability across the entire visible wavelength range (400‐700 nm); (2) optoelectronic performance metrics (photoluminescence quantum yield (PL QY) > 90%, full‐width at half‐maximum (FWHM) < 40 nm) approaching those of conventional Cd/Pb‐based QDs [23, 24, 25, 26, 27, 28, 29, 30]; and (3) being free of heavy metal toxicity, in compliance with the restriction of the use of certain hazardous substance (RoHS)‐compliant composition [31]. Such remarkable characteristics establish InP‐based QDs as the preeminent candidate for sustainable next‐generation display technologies in an increasingly eco‐conscious global market.

**FIGURE 1:**
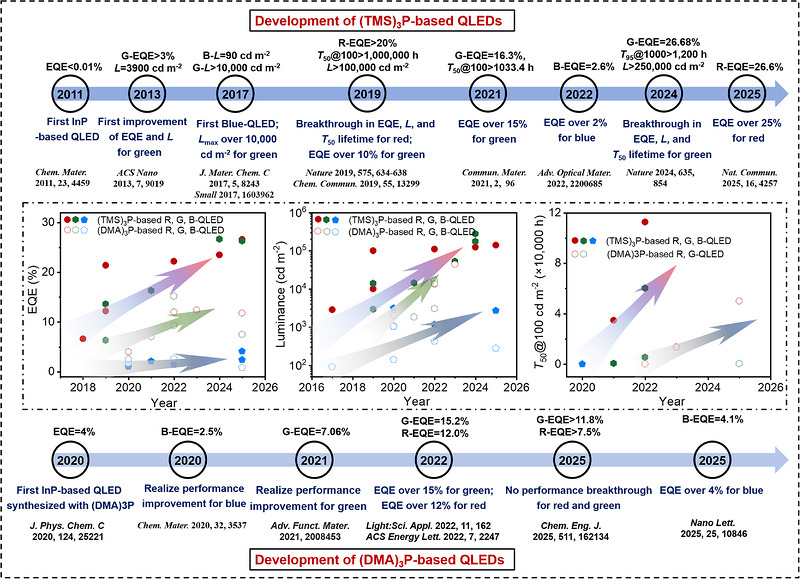
Development of (TMS)_3_P and (DMA)_3_P‐based QLEDs.

As a highly reactive phosphorus precursor, tri(trimethylsilyl)phosphine ((TMS)_3_P) has become one of the most widely utilized reagents for synthesizing high‐quality InP‐based QDs. Recent advances in synthetic methodology refinement, core/shell structure engineering, along with precise surface chemistry control and defect passivation techniques have led to remarkable enhancements in the optoelectronic properties of red‐ and green‐emitting InP‐based QDs and their corresponding QLEDs [23, 24, 28, 29, 32, 33]. Currently, the green and red InP‐based QLEDs have achieved a high EQE of over 20%, a high luminance of over 100,000 cd m^−2^, and a long *T*_95_ lifetime (time to 95% initial luminance retention) of over 1,200 h [23, 24]. These dual advancements in both efficiency and device lifetime collectively represent a transformative leap forward for InP‐based QLED technology. Nevertheless, the synthesis of these high‐performance InP‐based QDs mainly depends on (TMS)_3_P, which presents multiple drawbacks, including high cost, flammability/explosiveness, and generation of toxic PH_3_ when exposed to air, severely hindering both fundamental research and industrial applications. In recent studies, some performance improvements in QDs and QLEDs using safer and more cost‐effective tris(dimethylamino)phosphine ((DMA)_3_P) have been demonstrated [26, 34, 35, 36]. For instance, in 2022, Zhao et al. demonstrated a peak EQE of 15.2% for green InP‐based QLEDs by inserting the gradient inner shell layer ZnSe_x_S_1‐x_. The gradient inner shell to diminish the interface defects upon balancing the lattice mismatch and promoting the balanced injection of the carriers by lifting the conduction band (CB) and valence band (VB) of InP‐based QDs [35]. However, as shown in Figure 1, (DMA)_3_P‐based QDs and their corresponding QLEDs still exhibit substantially inferior efficiency, luminance, and operational lifetime compared to (TMS)_3_P‐based counterparts.

Previous reviews about InP‐based QDs and QLEDs mainly focused on the QDs synthesized with (TMS)_3_P, though some achievements about InP‐based QDs synthesized with (DMA)_3_P were also mentioned [37, 38, 39, 40, 41, 42, 43, 44]. A systematic analysis and understanding of the inferior performance of (DMA)_3_P‐based QDs and their electroluminescent devices remains notably absent. Therefore, this review began with a molecular‐level analysis of the fundamental differences between (TMS)_3_P and (DMA)_3_P, thereby elucidating their distinct influences on the nucleation mechanism of InP. Subsequently, we identified the key challenges to InP core synthesized with (DMA)_3_P, specifically their susceptibility to oxidation and poor exciton confinement. To address these issues, we proposed several strategies, including modulation of the InP core, the design of a core/shell structure, and engineering of the surface ligands. Furthermore, we investigated the performance limitations of QLEDs based on (DMA)_3_P‐synthesized InP‐based core/shell QDs, and proposed enhancement strategies that primarily address charge transport modulation and charge leakage suppression. Finally, we analyzed the key bottlenecks hindering the further development of these materials and devices, while proposing potential solutions and future research directions to overcome these limitations.

## 2 Comparison Between (TMS)_3_P and (DMA)_3_P in the Synthesis of InP‐Based QDs

### 2.1 Fundamentals About the Two Phosphorus Sources

The synthesis of reasonably sized and well‐dispersed InP‐based QDs was first achieved in the 1990s [45], yet subsequent progress in producing high‐quality InP‐based QDs has been notably slow. As Angelé et al. highlighted the primary challenges in InP‐based QDs preparation stem from the inherent reactivity of phosphorus precursors, which often renders the synthesis process difficult to control [46]. Various phosphorus sources have been explored in previous studies, including (TMS)_3_P [23, 24, 26], (DMA)_3_P [28, 35, 36], elemental phosphorus (P_4_) [47], gaseous phosphine (PH_3_) (generated from calcium phosphide or zinc phosphide) [48], all‐inorganic solid metal phosphorus (NaOCP) [30], PCl_3_ [49], Tri(pyrazolyl)phosphanes [50], tris(hexylthio)phosphine [51], (Na/K)_3_P [52], etc. Currently, the synthesis of high‐quality InP‐based QDs predominantly relies on two main phosphorus precursors: (TMS)_3_P and (DMA)_3_P (Table 1). These precursors exhibit markedly different chemical behaviors that profoundly influence InP core formation.

**TABLE 1:** Comparative performance of phosphorus sources.

Precursor	Molecular structure	Toxicity	Reactivity	Cost	Handling
(TMS)_3_P	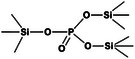	Moderate	High	High	Sensitive to moisture
(DMA)_3_P	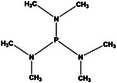	Low	Moderate	Low	Easy

The main advantage of (TMS)_3_P lies in its appropriate electronic structure, which enables the release of P^3^^−^ ions without requiring oxidation or reduction. Additionally, (TMS)_3_P derives its stability from the electron‐donating effect of trimethylsilyl (Si(CH_3_)_3_) groups, where the low electronegativity of silicon facilitates charge delocalization. The relatively weak Si‐P bonds (approximately 70–80 kcal mol^−1^) enable facile liberation of reactive phosphorus species through either nucleophilic substitution or β‐hydride elimination pathways. While this property enhances reaction kinetics, it frequently results in uncontrolled burst nucleation phenomena [53]. Consequently, the stringent regulation of both thermal and concentration parameters becomes imperative to achieve narrow size dispersity. Furthermore, the inevitable formation of silicon‐based decomposition products raises concerns about potential lattice incorporation, which may detrimentally affect charge carrier dynamics in the resulting QDs. From a practical standpoint, (TMS)_3_P presents substantial handling challenges due to its extreme hydrolytic instability. Upon atmospheric exposure, instantaneous oxidation yields phosphate and siloxane derivatives, while aqueous contact generates highly toxic PH_3_ gas, posing significant challenges for safe handling, storage, and transportation [53, 54].

Conversely, (DMA)_3_P demonstrates superior synthetic controllability attributable to its distinctive electronic structure. The dimethylamino substituents function as potent σ‐donors through nitrogen lone pair donation, establishing robust P‐N dative bonds (bond energy ∼90‐100 kcal mol^−1^) that require significant activation energy for dissociation. This kinetic barrier effectively regulates the release rate of phosphorus, effectively decoupling nucleation from growth phases. Such controlled release characteristics prove indispensable for achieving monodisperse size distributions, particularly when coupled with hot‐injection techniques. However, the P in (DMA)_3_P is in the +3 oxidation state, a limitation that necessitates the use of additional reduction agents (such as oleylamine) to initiate the reaction [55, 56]. In addition, the ancillary benefit of generating exclusively volatile dimethylamine byproducts (HN(CH_3_)_2_) permits efficient in situ removal through mild inert gas flow, eliminating potential surface contamination and ensuring stoichiometric purity. These combined attributes position (DMA)_3_P as the precursor of choice for precision synthesis of optoelectronically graded InP nanocrystals, notwithstanding its marginally higher thermodynamic instability compared to silyl‐phosphine analogs [57, 58]. In addition, recent advances in mechanistic understanding further suggest that the dimethylamino ligand may participate in stabilizing intermediate species during QD growth, offering additional avenues for synthetic refinement [55].

### 2.2 Impacts of Phosphorus Sources on the Nucleation of InP

**FIGURE 2:**
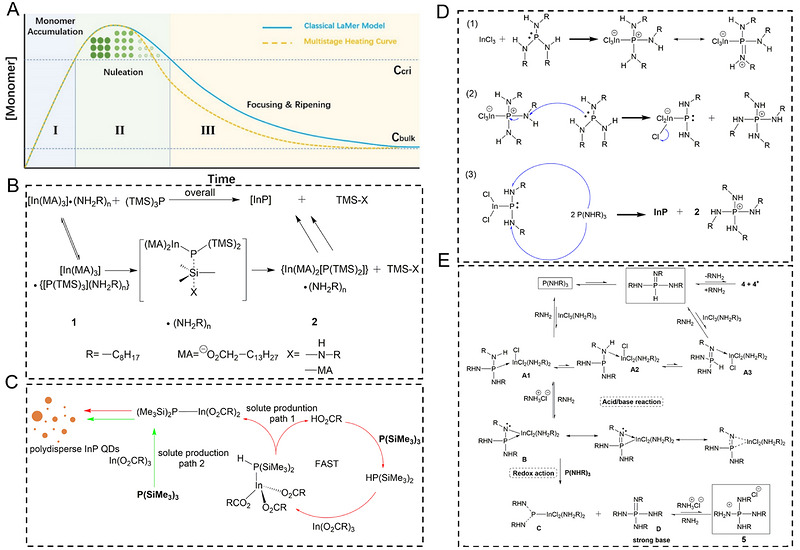
(A) LaMer model of nanocrystal nucleation and growth. Reproduced with permission [59]. Copyright 1950, American Chemical Society. (B) Mechanistic pathway for amine‐inhibited InP synthesis. Reproduced with permission [61]. Copyright 2010, John Wiley & Sons. (C) Precursor conversion reactions leading to colloidal InP nanocrystals based on (TMS)_3_P. Reproduced with permission [62]. Copyright 2013, American Chemical Society. (D) Chemical reaction mechanism for (DMA)_3_P‐based InP synthesis. Reproduced with permission [55]. Copyright 2016, American Chemical Society. (E) Mechanism for the first In‐P bond formation based on (DMA)_3_P. Reproduced with permission [63]. Copyright 2016, American Chemical Society.

The synthesis of high‐performance InP cores with uniform size lies in the precise control over nucleation and growth dynamics. The classical nucleation and growth kinetics model, introduced by LaMer et al. in 1950, offers fundamental insights into the formation of monodisperse particles (Figure 2A) [59]. According to this model, the synthesis proceeds through three stages: the first stage is precursor conversion, in which the precursors rapidly decompose into reactive monomers, leading to a sharp increase in monomer concentration. Once the monomer concentration surpasses the critical supersaturation threshold, the second stage of nucleation occurs, abruptly depleting the monomer concentration. With monomers rapidly consumed during nucleation, their concentration soon becomes insufficient to sustain new nucleation events, marking the transition into the third stage of growth. Here, the monomer concentration dictates whether the system undergoes size focusing (narrowing size distribution) or Ostwald ripening (broadening size distribution).

However, for InP‐based QDs, the high reactivity of the phosphorus precursor results in instantaneous nucleation upon injection, making it challenging to decouple nucleation from subsequent growth [60]. Research has revealed that InP growth proceeds through an intermediate stage involving magic cluster intermediates [56, 61, 62, 63]. Therefore, investigating the chemical mechanisms—including precursor transformation kinetics and reaction pathways—provides deeper insight into the factors beyond reactivity that directly influence polydispersity.

For (TMS)_3_P, in 2010, Bawendi and coworkers proposed a reaction mechanism for the synthesis of InP‐based QDs through the reaction of In(MA)_3_ with (TMS)_3_P at elevated temperatures. According to their proposed mechanism, the process initiates with the coordination of In(MA)_3_ to Lewis bases, such as octylamine, in the outer solvation sphere. The reaction proceeds through a reversible first step where one (TMS)_3_P molecule enters the solvation sphere, forming an intermediate complex (complex 1). Subsequently, complex 1 undergoes an irreversible transformation involving the dissociation of a myristate ligand, the formation of a stable In‐P bond, and the elimination of a TMS group, yielding a molecular intermediate (complex 2). This intermediate then serves as the precursor for the formation of [InP] clusters and ultimately evolves into InP nanocrystals (Figure 2B). The authors found that the phosphorus precursor molecule was completely depleted after nucleation, thus allowing the subsequent QDs growth process to be entirely dominated by the maturation of the nonmolecular InP cores, leading to a broader size distribution of InP‐based QDs [61]. This conventional understanding was challenged in 2013 by Cossairt, who identified a competitive acid‐catalyzed pathway during (TMS)_3_P conversion to InP that adversely affected monodisperse core formation. In this alternative reaction, proton‐donating species (e.g., myristic acid, H_2_O, or MeOH) facilitate (TMS)_3_P protonolysis, yielding the secondary phosphine HP(SiMe_3_)_2_. Rapid injection of these phosphorus precursors into heated (∼315°C) In^3^^+^‐containing solutions initiates a reaction between the indium centers and phosphorus species (as P^3^^−^ or HP^2^^−^), producing InP monomers. Once monomer concentration exceeds the critical supersaturation threshold, the nucleation process commences spontaneously. Most notably, primary amine ligands (e.g., n‐octylamine) were found to modulate reaction kinetics through indium coordination, simultaneously retarding In‐P bond formation while ensuring controlled core nucleation. Conversely, excess proton source (particularly myristic acid) accelerates (TMS)_3_P decomposition, generating high concentrations of reactive phosphorus intermediates such as HP(SiMe_3_)_2_ (Figure 2C). This leads to uncontrolled nucleation kinetics and the formation of polydisperse cores [62].

The reaction principles of (TMS)_3_P and (DMA)_3_P differ greatly due to their distinct molecular structures. Early mechanistic studies on (DMA)_3_P‐based synthesis suggested that oleylamine—serving as both solvent and ligand—could release labile protons (H^+^) to activate (DMA)_3_P, forming reactive PH_3_ intermediates. As proposed by Song et al., this process was thought to trigger rapid nucleation via reaction with In‐oleylamine complexes, enabling the production of high‐quality InP‐based QDs [56]. However, this hypothesis faces thermodynamic limitations: the primary amine (–NH_2_) of oleylamine has a high proton affinity, and its deprotonation would demand exceptionally harsh conditions (e.g., strong base or extreme energy input), which are inconsistent with typical synthesis environments. Subsequent work by Tessier, Buffard, and colleagues resolved this discrepancy by introducing a more feasible mechanism, fundamentally revising the understanding of InP formation. Their studies revealed that under heating, (DMA)_3_P undergoes an amine exchange reaction with oleylamine, yielding P(NHR)_3_ (where R represents oleylamine's alkyl chain) while releasing volatile dimethylamine (HNMe_2_). The resulting P(NHR)_3_ then coordinates with InCl_3_ to form a reactive intermediate, which subsequently participates in a redox process to generate InP cores. Notably, for each InP (P: ‐III), four P(NHR)_3_ molecules are consumed—three oxidized to P(V) phosphonium salts (e.g., [P(NHR)_4_]^+^Cl^−^) and one reduced to P(‐III) for lattice incorporation [55, 63]. However, Tessier and Buffard et al. proposed different theoretical explanations for the reaction pathway. Tessier et al. suggested that aminophosphine serves a dual function, acting as both a phosphorus precursor and a reducing agent. Their proposed stoichiometry (4 P(+III) → P(‐III) + 3 P(+V)) suggests the simultaneous formation of InP and phosphonium salt (P(NHR)_4_Cl) byproducts. This pathway is substantiated by robust experimental evidence, including ^3^ ^1^P NMR spectroscopy and mass spectrometry data that clearly identify P(NHR)_4_^+^ as the dominant reaction byproduct (Figure 2D) [55]. In contrast, Buffard et al. proposed a reduction mechanism based on proton‐transfer and phosphorus hypervalency‐based. They argued that the change in phosphorus oxidation state does not occur via simple disproportionation, but rather involves multi‐step proton transfer and nucleophilic attack. And they emphasized that primary amines (such as oleylamine) are not only solvents, but also participate in proton transfer, promoting the formation of P‐In bonds (Figure 2E) [63].

While these models present different views of the underlying chemistry, both research groups have significantly advanced our understanding of the intricate reaction dynamics in colloidal InP synthesis. These mechanistic insights provide valuable guidance for future synthetic optimization and material design.

## 3 Advances Achieved for (DMA)_3_P‐Based InP QDs

Compared to bulk materials, QDs in the size range of 0–20 nm exhibit distinct optoelectric properties due to the quantum confinement effect. In particular, the confinement energy, the quantum energy of localization for electrons and holes, becomes more prominent as the spherical size of QDs decreases and approaches the Bohr radius. For InP‐based QDs, their small effective electron mass (0.08 m_0_) combined with a high dielectric constant (ε = 12.9) promotes extensive electron delocalization, yielding an expansive exciton Bohr radius of about 10 nm [64, 65]. Meanwhile, with a bulk bandgap of 1.35 eV (corresponding to a ∼920 nm wavelength), InP‐based QDs exhibit size‐tunable emission spanning the entire visible spectrum (blue to deep red) through quantum confinement effects. Additionally, the superior carrier mobility of InP relative to other semiconductor materials positions these QDs as promising candidates for high‐performance optoelectronic devices.

Given these advantageous properties, understanding and controlling the recombination pathways of charge carriers is critical for realizing the potential of InP‐based QDs in practical applications. Upon photoexcitation with energy above the bandgap of InP‐based QDs, an electron in the valence band absorbs a photon and is promoted to an excited state. Following energy relaxation to the ground state, the recombination of the electron‐hole can produce luminescence. However, the electrons in QDs also can undergo a relaxation process to the ground state without any luminescence via non‐radiative recombination, such as Shockley‐Read‐Hall recombination [66] and field‐induced quenching [67]. Since the radiative recombination competes with these non‐radiative recombination processes, the PL QY of QDs can be described as PL QY = (1−N) k_r_/(k_r_ + k_nr_), where N is the proportion of QDs in non‐emitting states, k_r_ is the radiative recombination rate, and k_nr_ is the non‐radiative recombination rate. Therefore, to enhance radiative recombination, it is essential to engineer the core to suppress defects in the interface of core/shell, optimize the core/shell structure for effective charge carrier confinement within the core, and modulate surface ligands to improve the stability of core/shell QDs [23, 24, 28, 35, 68].

### 3.1 Understanding of the Key Challenges for InP‐Based QDs

**FIGURE 3:**
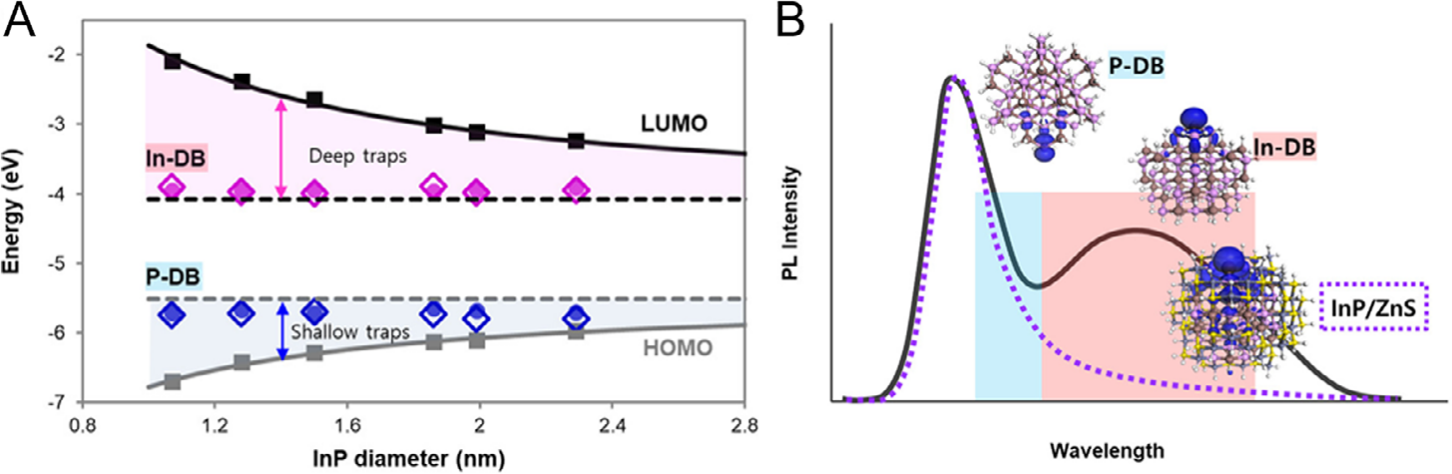
(A) Energy levels of HOMO, LUMO, In‐DBs, and P‐DBs versus InP diameter calculated by DFT and (B) schematic figures of band‐edge emission and defect emission in PL spectra. Reproduced with permission [40]. Copyright 2020, American Chemical Society.

The covalent character of the In‐P bond (bond covalency: 0.42 vs. CdSe: 0.72) enhances optical stability compared to ionic II‐VI QDs. However, InP‐based QDs exhibit smaller core sizes compared to Cd‐based QDs for the same wavelength emission [69, 70]. Consequently, their high surface‐to‐volume ratio combined with the smaller effective electron mass of InP (0.08 m_0_), renders the electrons particularly vulnerable to surface defects and dangling bonds [71]. Figure 3A presents the energy levels of surface defects and band edges as a function of InP cluster size based on density functional theory (DFT) calculations. With increasing the InP cluster size, the highest occupied molecular orbital (HOMO) level rises and the lowest unoccupied molecular orbital (LUMO) level decreases due to the quantum confinement effect. In contrast, the defect levels of indium dangling bonds (In‐DB, ‐3.947 eV) and phosphorus dangling bonds (P‐DB, −5.717 eV) remain independent of QD size. It was observed that the In‐DB can exceed the LUMO in energy for QDs larger than 11.0 nm in diameter, whereas the P‐DB aligns with the HOMO near a diameter of 4.7 nm. Based on the energy differences between the In‐DB and LUMO and between the P‐DB and HOMO, the In‐DB acts as a deep trap and the P‐DB as a shallow trap in the PL spectra, as illustrated in Figure 3B [40]. Additionally, the surface P atoms exhibit particular susceptibility to oxidation due to the high oxophilicity of group III and V elements, forming polyphosphates and orthophosphates through reactions with ambient oxygen. This oxidation process not only introduces non‐radiative recombination centers, but also substantially degrades PL QY [69, 72, 73, 74]. Hence, this comprehensive understanding of InP QDs' fundamental properties highlights both their significant potential for optoelectronic applications and the critical need for surface engineering to mitigate defect‐related performance limitations.

The inherent instability of QDs is a critical factor undermining the overall device instability, largely due to unintended defects that occur within the particles. Compared to Cd‐based QDs, InP‐based QDs exhibit a higher propensity for defect formation during synthesis. Furthermore, degradation caused by material oxidation, which occurs during or after synthesis as a result of interactions with the external environment, is a key contributor to QD instability. To address these challenges, the following discussion will focus on strategies to enhance the optoelectronic performance of InP‐based QDs, including InP core modification, core/shell structure design, and surface ligand engineering [75, 76, 77, 78, 79].

### 3.2 Modulation of (DMA)_3_P‐Based InP Cores

The performance of QDs is significantly constrained by surface defects and oxidation issues of the core, which impede further enhancement of their properties. These imperfections introduce non‐radiative recombination centers that diminish PL QY, promote fluorescence quenching, and undermine the stability of the QDs under ambient or operational conditions. Furthermore, surface oxidation, often resulting from exposure to air or moisture, leads to the degradation of the core material, altering its electronic structure and thereby deteriorating its optical characteristics over time. Hence, core modification—encompassing oxide removal and surface passivation—has emerged as a pivotal strategy for improving the optical performance of QDs.

#### 3.2.1 Removing Dangling Bonds on InP Core

**FIGURE 4:**
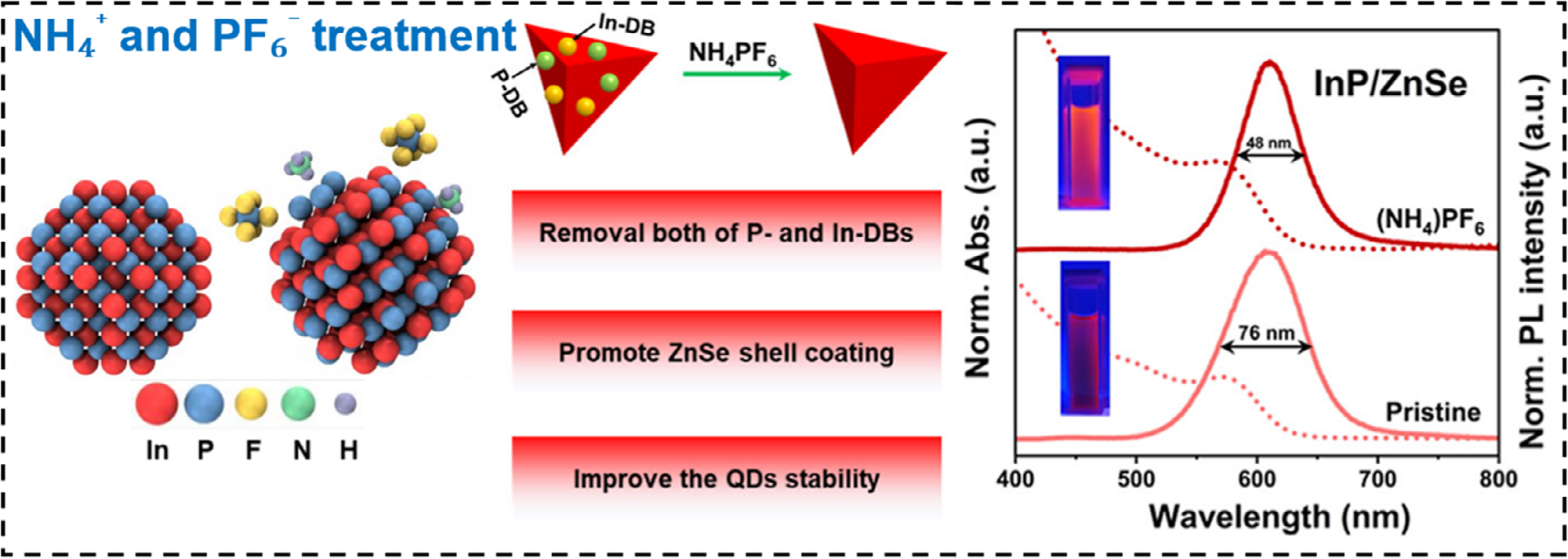
Schematic diagram of NH_4_PF_6_ function (left). Absorption and PL spectra of NH_4_PF_6_‐treated InP/ZnSe QDs and the pristine one (right). Reproduced with permission [80]. Copyright 2024, American Chemical Society.

Synergistic passivation of both In and P dangling bonds is crucial for significantly enhancing the optoelectronic performance and stability of QDs. Sun et al. developed a novel one‐step surface treatment strategy for simultaneous peeling and passivation of both In‐ and P‐DBs in (DMA)_3_P‐based InP QDs via introducing inorganic pseudohalogen ammonium salt. In the OAm solution, NH_4_PF_6_, as a kind of ionic compound, easily decomposes NH_4_^+^ and PF_6_^−^ ions. The resulting NH_4_^+^ and PF_6_^−^ ions selectively passivate the In‐DBs and P‐DBs on the surface of InP cores, forming P‐NH_4_ and In‐PF_6_ bonds, thereby simultaneously eliminating both In and P defects. Additionally, owing to the similar ionic radius and chemical behaviors as halides, PF_6_^−^ anions can substitute halides to passivate the surface defects and improve the chemical stability of the InP core (Figure 4) [80]. This synergistic approach achieves comprehensive surface reconstruction through simultaneous cation‐anion cooperative action, addressing both types of dangling bonds in a single treatment step.

#### 3.2.2 Suppressing Surface Oxide on InP Cores

Fluoride treatment serves as a critical technique for optimizing III‐V group QDs (e.g., InP). Research demonstrates that fluorides can effectively eliminate surface oxides (such as InPO_4_ and P_2_O_7_) from QDs, thereby reducing non‐radiative recombination centers and enhancing PL QY [23, 78, 79, 81, 82, 83, 84, 85]. Fluoride treatment offers distinct advantages due to the high electronegativity and compact atomic size of fluorine, which enables effective surface passivation while simultaneously modulating band‐edge potentials, thereby significantly enhancing the air stability of QDs.

According to literature reports, fluoride treatment can be divided into HF treatment, in situ HF generation method, and anhydrous ammonium fluoride method [26, 34, 36, 79, 84]. For instance, Sun et al. achieved a breakthrough in the synthesis of (DMA)_3_P‐based red InP QDs, demonstrating a record‐high PL QY of 97.7%. This exceptional performance was realized through HF‐assisted processing, in which HF reacts with InPO_4_ to form soluble InF_3_ and H_3_PO_4_, while F^−^ passivates In‐DBs, thereby reducing surface defect states and enhancing PL efficiency [84].

**FIGURE 5:**
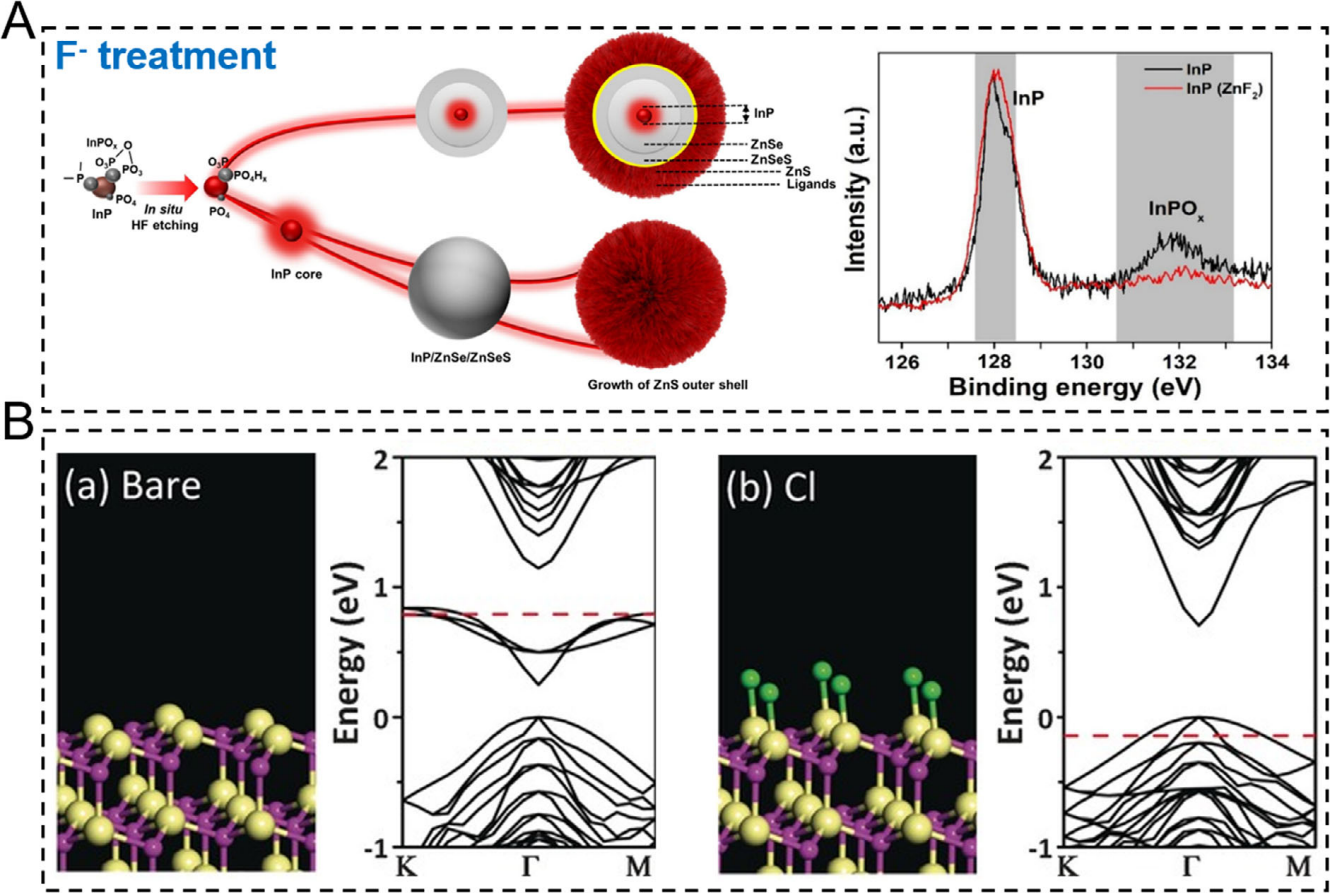
(A) Schematics of multiple‐shell InP QD synthesis and in situ HF etching (left). P2p peaks of the InP cores with and without in situ HF etching (right). Reproduced with permission [36]. Copyright 2025, Elsevier. (B) DFT calculations for indium‐terminated InP (111) surfaces of InP bare core and Cl‐passivated InP core. Reproduced with permission [88]. Copyright 2016, John Wiley & Sons.

However, HF is highly corrosive and poses severe safety risks—skin contact or inhalation of its vapors can cause critical injuries or even fatalities. During high‐temperature synthesis, HF gas may rapidly increase the internal pressure of the reaction system, creating an explosion hazard. Additionally, HF etching requires precise control of concentration and duration, as excessive etching can damage the InP core structure, leading to non‐uniform QD sizes or an increase in surface dangling bonds. Therefore, developing a milder etching strategy is essential. Oh et al. developed a safer, oxygen‐free synthetic strategy for high‐quality InP‐based QDs using (DMA)_3_P as the phosphorus precursor and ZnF_2_ as a reactive additive in an alkylamine solvent. As can be seen from Figure 5A, the in‐situ generation of HF from ZnF_2_ effectively suppresses the formation of polyphosphates (P_2_O_7_ˣ^−^) and mixed oxides (InPO_x_), facilitating the controlled growth of red and green‐emitting core/shell QDs. This approach yields enhanced PL QYs of 93% and 88% for red and green QDs, respectively [36]. Additionally, Owen and Reiss et al. reported a safer way of generating HF in situ using benzoyl fluoride in the presence of octylamine and successfully removed surface defects and increased the PL QY of the InP core to over 80% (the highest reported PL QY for InP QDs without a shell) [26, 34].

However, most of the above reaction systems contain carboxylic acids. Nayral, Delpech, et al. have demonstrated that at elevated temperatures, carboxylic acids could undergo a competing side reaction that generates dialkylketone (palmitone) with the release of water. This side reaction is undesirable because the generated water could induce partial oxidation of the InP core surface, leading to the formation of an InP/InPO_x_ core/shell heterostructure. This oxidation not only degrades the optical properties of the InP core but also disrupts subsequent shell growth [73, 86, 87, 88, 89]. To address this challenge, Jeong et al. demonstrate a facile acid‐free synthesis of highly crystalline InP‐based QDs with unique tetrahedral morphology. The approach utilizes (DMA)_3_P and indium trichloride as phosphorus and indium precursors, respectively, dissolved in oleylamine. Comprehensive chemical analyses reveal that both oleylamine and chloride ligands cooperatively stabilize the tetrahedral InP QDs, which are predominantly enclosed by cation‐rich (111) facets. As shown in Figure 5B, DFT calculations suggest that the fractional dangling electrons of the In‐rich (111) surface can be fully passivated through a halide‐amine co‐passivation mechanism, with three halide ligands and one primary amine ligand per (2 × 2) surface unit effectively satisfying the 8‐electron rule. This innovative ligand strategy provides a promising pathway for the controlled synthesis of stable III‐V QDs with well‐defined surfaces [88]. In addition, zinc carboxylate, a common shell precursor, can also generate water molecules as a byproduct. This often induces the formation of phosphate layers on the surface of the InP core, which, in turn, leads to non‐uniform shell growth. To avoid the use of zinc carboxylate, Long et al. developed an acid‐free approach. They employed zinc chloride as the zinc precursor and oleylamine as the sole solvent, and through careful optimization of the shell structure, successfully synthesized InP‐based QDs with a record‐high PL QY (96%) and the narrowest reported FWHM (41 nm) at the time [89].

#### 3.2.3 Surface Ligand Engineering of InP Core

**FIGURE 6:**
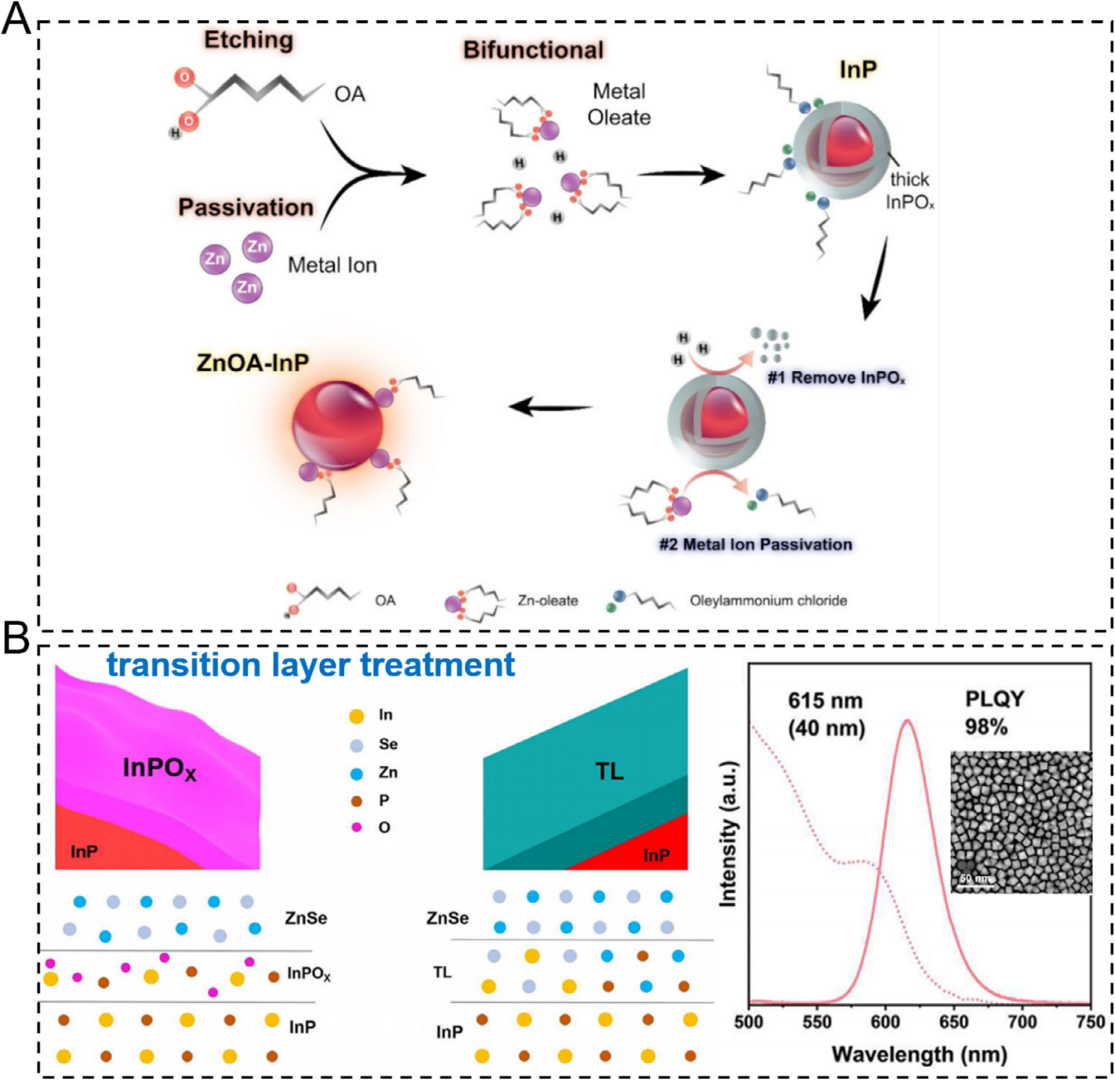
(A) Schematic diagram of the function of metal oleate on InP core: passivate InP surface and etch surface oxide layer. Reproduced with permission [91]. Copyright 2022, MDPI. (B) Schematic diagram of interface engineering between the core/shell interface (left). Absorption and PL spectra of InP/TL/ZnSe/ZnS QDs (right). Reproduced with permission [77]. Copyright 2024, American Chemical Society.

Significant progress in InP core synthesis has also been achieved through innovative surface engineering and interface design. For instance, Jiang and colleagues demonstrated precise control over QD size distribution through the strategic introduction of ZnX_2_ during the intermediate synthesis stage. The authors proposed that ZnX_2_, binding to the QD surface functions, as a Z‐type ligand, which passivates surface defects—thereby suppressing defect‐state emission—while simultaneously suppressing Ostwald ripening processes [90]. In a separate study, Chen et al. occasionally discovered that a higher‐concentration oleic acid (OA) in toluene could partially passivate the surface of (DMA)_3_P‐based InP QDs, though the efficiency of this method remained limited. To overcome this constraint, they developed an advanced passivation strategy involving metal oleates with dual etching‐passivation functionality. As shown in Figure 6A, in this system, oleic acid provides protons to dissolve surface oxides, while simultaneously, both metal ions and oleate ligands contribute to the effective passivation of the InP surface, further enhancing the optical and structural properties of the QDs [91].

Parallel developments by Tian's group showcased the synthesis of high‐performance aminephosphine‐based InP/ZnSe/ZnS QDs through the innovative incorporation of an alloyed oxidation‐free In‐ZnSe transition layer (TL) at the core/shell interface. The engineered TL not only has the essential function of preventing oxidation of the core and relieving interfacial strain, but also results in oriented epitaxial growth of the shell. Most notably, this interfacial engineering approach dramatically reduces non‐radiative recombination at core/shell interfacial defect states, culminating in exceptional PL QY reaching 98% (Figure 6B) [77]. Also, recent studies have demonstrated that the introduction of a sterically hindered diamine (DIBA) effectively modulates the size and optical characteristics of InP QDs. DIBA interacts with the phosphorus precursor to form an intermediate species, slowing reaction kinetics and thereby controlling QD nucleation and growth rates. Furthermore, precise tuning of DIBA concentration allows for size‐controlled synthesis, yielding InP/ZnS core/shell QDs with tunable PL emissions spanning from 480 nm to 620 nm [92].

The optical properties and stability of InP‐based QDs have been significantly enhanced through core modifications involving surface passivation, oxide removal, and ligand engineering. However, due to their large ratio of surface area to bulk, non‐radiative recombination at surface sites competes very efficiently with luminescence. To separate charge carriers from their surroundings and confine them in the core as much as possible, an epitaxially growing shell of a wide bandgap to form type‐I QDs is necessary. This shell effectively isolates the core from the surrounding environment, suppresses surface trapping states, and enhances charge carrier confinement, resulting in higher emission efficiency and improved resistance to photobleaching [23, 24, 28, 35]. The following section will focus on the design of core/shell structures and the modulation of the bandgap.

### 3.3 Core/Shell Structure Design for (DMA)_3_P‐Based InP QDs

In 1994, Micic et al. pioneered the colloidal synthesis of InP‐based QDs, representing a major advancement in III‐V semiconductor nanocrystal research. However, these early InP cores exhibited limited practical utility due to their low PL QY and polydisperse size distribution [45]. A critical improvement came in 2001 when Haubold et al. introduced the first InP/ZnS core/shell QDs, boosting the PL QY from below 1% to 23% while substantially enhancing stability. This work laid the foundation for subsequent refinements in core/shell QD design [93]. Despite these advances, the reliance on toxic and expensive (TMS)_3_P remained a key limitation. A breakthrough emerged in 2013 when Song et al. developed an alternative approach using (DMA)_3_P as a safer, more economical phosphorus precursor. Through precise control of reaction parameters—including Zn additives, temperature, and phosphorus stoichiometry—they achieved InP/ZnS QDs with a high PL QY of 51%–53% and a narrow emission bandwidth of 60–64 nm. Further optimization via secondary shell growth elevated the PL QY to 64%–68% while dramatically improving photostability [56]. This strategy not only circumvented the need for (TMS)_3_P but also established a scalable and environmentally benign route for high‐performance InP‐based QDs.

#### 3.3.1 Alloyed Core

**FIGURE 7:**
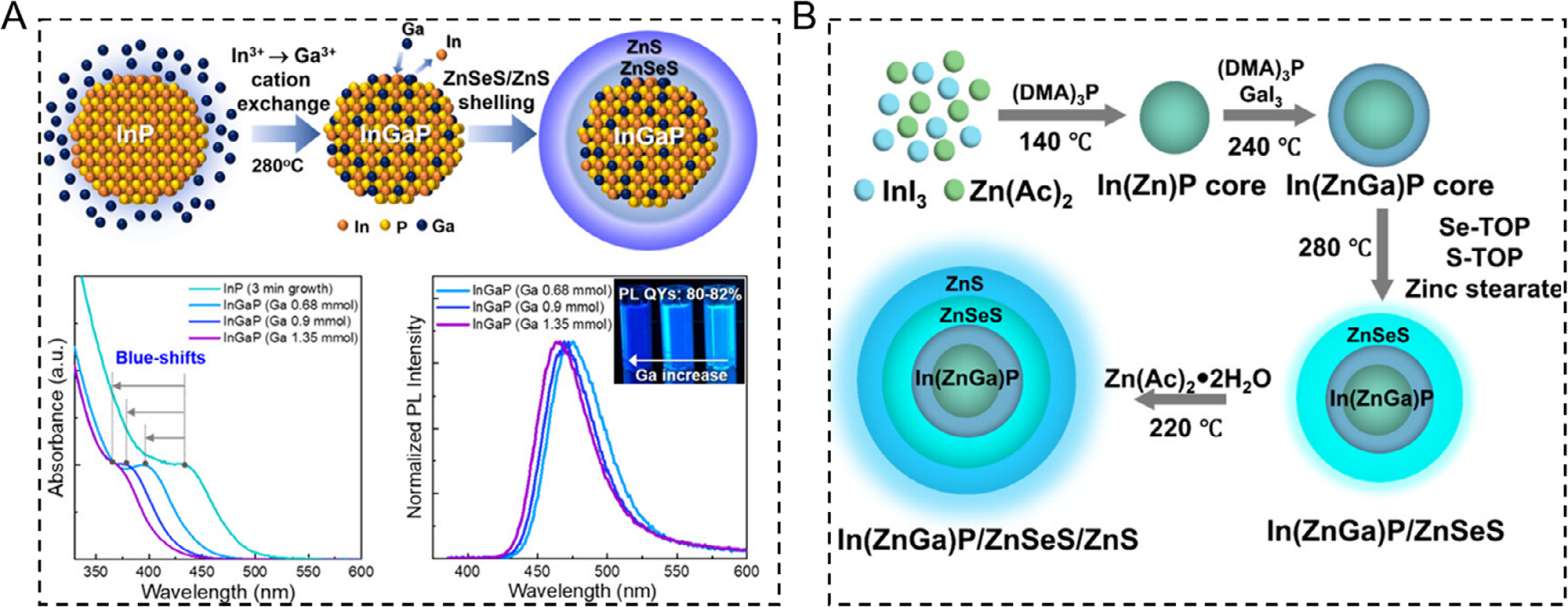
(A) Schematic illustration of In^3+^‐to‐Ga^3+^ cation‐exchange‐based InGaP core and subsequent ZnSeS/ZnS double shelling (top). Absorption and PL spectra of a series of cation‐exchanged InGaP QDs (bottom). Reproduced with permission [95]. Copyright 2020, American Chemical Society. (B) Synthesis process diagram of In(ZnGa)P/ZnSeS/ZnS QDs. Reproduced with permission [96]. Copyright 2024, American Chemical Society.

In addition to optimizing the surface of the core, core alloying presents an alternative approach for significantly improving its PL QY. A notable advancement was demonstrated by Jiang et al., who successfully incorporated Ga ions into (DMA)_3_P‐based red InP cores through thermally promoted cation exchange. The resulting Ga‐doped InP cores exhibited a remarkable improvement in PL QY, reaching up to 26%. Subsequent coating of these cores with ZnSeS and ZnS shells further boosted the PL QY to 62%, significantly outperforming the undoped core (36%) [94]. This work established Ga doping in InP cores as a promising strategy for enhancing the optical properties of InP‐based QDs. The synthesis of high‐performance blue‐emitting InP‐based QDs presents unique challenges due to the relatively low bulk bandgap (1.35 eV) of InP, particularly when targeting smaller core sizes required for blue emission. In this context, Ga doping has emerged as an effective approach. Yang et al. first synthesized the blue‐emissive ternary InGaP QDs through In^3+^‐to‐Ga^3+^ cation exchange strategy. As shown in Figure 7A, by precisely optimizing the GaI_3_ amounts and employing sequential passivation with ZnSeS inner and ZnS outer shells, they achieved consistent blue shifts in PL from 475 to 465 nm while maintaining exceptional PL QYs of 80–82% [95]. Despite these advances, the synthesis of pure blue‐emitting (450‐465 nm) InP‐based QDs relying solely on Ga doping remains challenging. A breakthrough was reported in 2024 with the development of a dual‐element modification strategy employing both Zn and Ga, in which the Zn element during nucleation inhibited the growth of the InP cores and narrowed the lattice while the Ga element further increased the material band gap by cation exchange (Figure 7B). Subsequent coating with ZnSeS and ZnS shells yielded deep blue‐emitting QDs at 457 nm with an outstanding PL QY of 84%, representing a significant advancement in the field of InP‐based blue QD synthesis [96].

#### 3.3.2 Single Shell

However, unprotected cores suffer from inherent limitations. Firstly, their surfaces are prone to interacting with ambient surroundings (e.g., oxygen and moisture), leading to instability in their chemical structure and optical properties. Secondly, their confined volume is insufficient for effective exciton confinement, causing some charge carriers to escape to surface defect states. This results in non‐radiative recombination, ultimately leading to a low PL QY and poor spectral stability. To achieve effective confinement of electron‐hole pairs within the core while minimizing stress‐induced lattice distortion, Shen et al. developed a multistep ZnS shell growth strategy to precisely control ZnS shell thickness. Their approach demonstrated that a second ZnS shell coating could produce a uniform, relatively thick shell while maintaining a high PL QY of 76.1% [97]. However, the excessive zinc stearate used during shell coating could lead to residual surface ligands that hinder carrier injection in QLEDs. Addressing this challenge, Sun et al. proposed an innovative solution by introducing zinc oleate and sulfur‐Tri‐n‐octylphosphine (S‐TOP) during the secondary ZnS shell growth. These additives not only facilitated epitaxial shell growth but also effectively reacted with residual zinc stearate. This dual‐function approach significantly improved device performance, increasing current density from 13 mA cm^−2^ to 121 mA cm^−2^ at 8 V in hole‐only devices. Consequently, the EQE was enhanced from 0.6% for InP/ZnS QLEDs to 1.7% for the optimized blue InP/ZnS/ZnS QLEDs [98].

#### 3.3.3 Double Shell or Alloyed Shell

**FIGURE 8:**
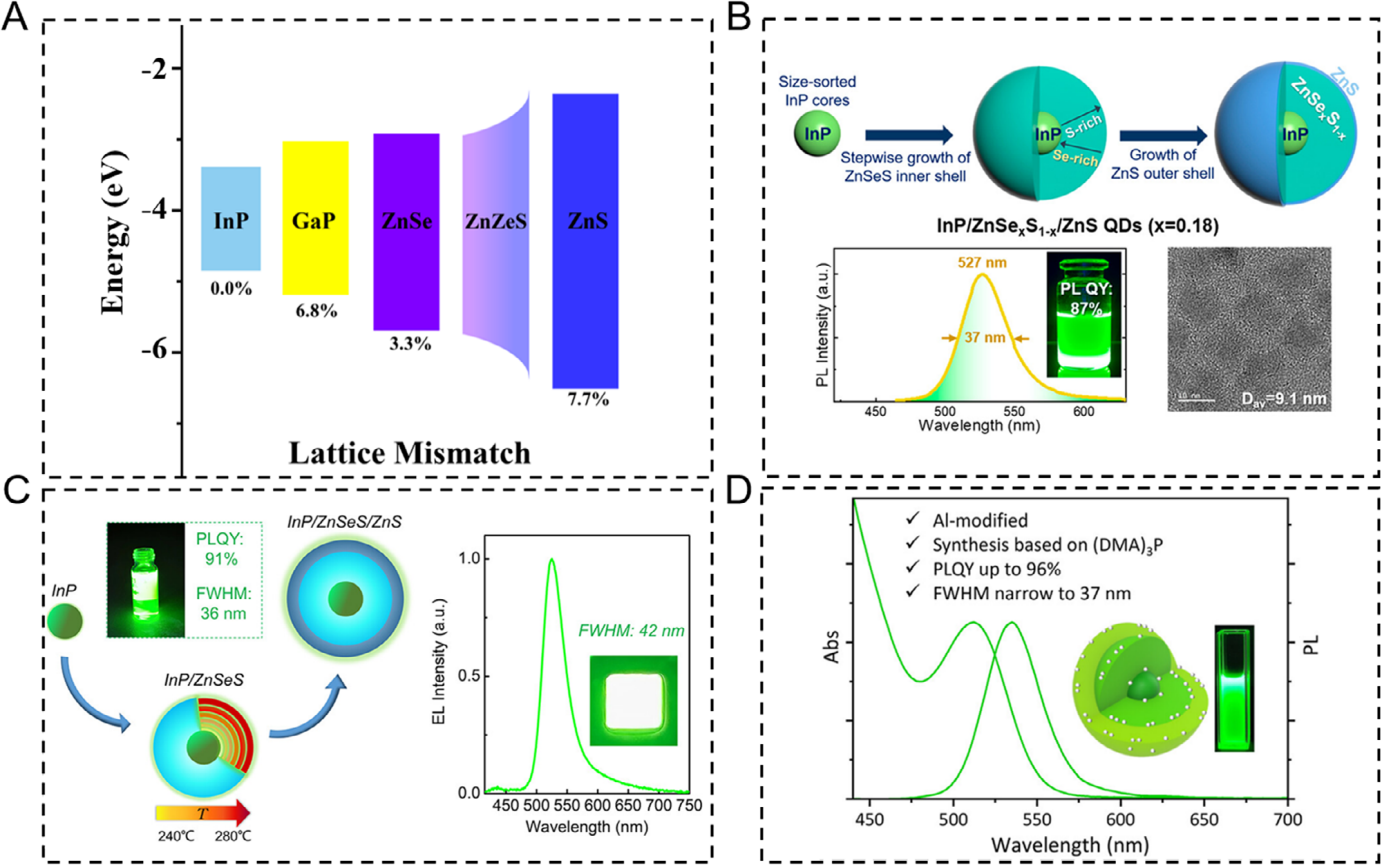
(A) Schematic diagram of energy level structure for InP, GaP, ZnSe, ZnSeS, ZnS. (B) The synthesis process of InP/ZnSe_x_S_1‐x_/ZnS QDs by the two‐step approach. PL spectra and TEM image of InP/ZnSe_x_S_1‐x_/ZnS QDs. Reproduced with permission [100]. Copyright 2020, American Chemical Society. (C) The synthesis process of InP/ZnSe/ZnS QDs via temperature‐gradient solution growth (left). PL spectra of InP/ZnSe/ZnS QDs (right). Reproduced with permission [101]. Copyright 2023, American Chemical Society. (D) Absorption and PL spectra of Al‐modified InP/ZnSeS/ZnS QDs. Reproduced with permission [58]. Copyright 2025, American Chemical Society.

Although a single shell can improve the stability and PL QY of QDs to some extent, the overall performance remains unsatisfactory. These limitations in PL performance are primarily attributed to significant interfacial strain caused by the substantial lattice mismatch (7.7%) between InP and ZnS [64]. Therefore, the design of the double shell is particularly crucial. The inner shell not only passivates surface defects of the InP core, but also alleviates the lattice mismatch between the InP core and the ZnS outer shell (7.7% for InP‐ZnS vs. 3.4% for InP‐ZnSe) through intermediate shell design. Additionally, the wide‐bandgap outer shell (e.g., ZnS) enhances exciton confinement, thereby improving PL efficiency [37, 64]. To mitigate the interfacial strain between InP and ZnS, researchers have introduced an intermediate inner shell with compositions such as ZnSe, ZnSeS, or GaP, prior to the deposition of the outer ZnS shell (Figure 8A) [35, 75, 76, 99]. This approach has proven effective, demonstrating notable improvements in PL QY and FWHM. For instance, Yang et al. reported red InP/ZnSe/ZnS core/shell/shell QDs with a relatively low FWHM of 44 nm along with a high PL QY of 86% by adopting an unconventional precursor of InBr_3_ instead of InCl_3_ and thickening the ZnSe inner shell [76]. Nevertheless, due to the interfacial strain between InP and ZnSe, the resulting QDs exhibited suboptimal performance, including a tetrahedral morphology accompanied by irregularly shaped large particles. To address these limitations, Tian et al. introduced an alloyed oxidation‐free In‐ZnSe TL at the core/shell interface. The TL not only has the essential function of preventing oxidation of the core and relieving interfacial strain but also promotes oriented epitaxial shell growth. The synthesized InP/TL/ZnSe/ZnS QDs had a high PL QY of 98%, a narrow FWHM of 40 nm, and a long biexciton Auger lifetime of 104 ps, demonstrating one of the best performances for InP‐based QDs synthesized by (DMA)_3_P [77].

While ZnSe/ZnS shell structures have been widely used for InP QDs, this structure presents several limitations, such as electron delocalization. The low energy barrier of ZnSe results in a wider distribution of electrons extending into the shell, which can promote unexpected destruction of the exciton, such as electron defects and Auger recombination. To better combine the advantages of ZnSe and ZnS shells, the design of component‐modulated ZnSe_1‐x_S_x_ inner shells needs to be considered. Yang et al. achieved a significant synthetic breakthrough via a two‐step approach, involving precise size fractionation of an as‐grown InP core followed by sequential deposition of a composition‐gradient ZnSe_x_S_1‐x_ inner shell and a ZnS outer shell. As can be seen from Figure 8B, by carefully optimizing the Se/S ratio, the resulting InP/ZnSe_x_S_1‐x_/ZnS QDs exhibit exceptional green (527 nm) photoluminescence features of a sharp FWHM of 37 nm and a high PL QY of 87% [100]. Building on this work, Sun et al. developed an alternative shell engineering strategy using sequential precursor injection to create a graded ZnSe(inner)‐ZnS(outer) intermediate shell layer. This approach further improved the green emission characteristics, achieving an outstanding PL QY of 95% [75]. Generally, growing the shells (especially the outer ZnS layer) at higher temperatures seems to be conducive to high‐quality epitaxy. While higher temperatures generally promote better crystalline growth, they can simultaneously have a negative impact on the highly active InP core. To resolve this trade‐off, Li et al. introduced a one‐pot synthesis strategy incorporating a temperature‐gradient solution growth (TGSG) method for the inner shell layer (240°C–280°C). The TGSG‐optimized InP/ZnSeS/ZnS multishell QDs showed superior performance (PL QY of 91%, FWHM of 36 nm) compared to those grown at fixed temperatures of 240°C (PL QY of 68%, FWHM of 40 nm) or 280°C (PL QY of 82%, FWHM of 45 nm), demonstrating the effectiveness of temperature‐controlled shell growth (Figure 8C) [101]. At the same time, alternative shell engineering approaches have also been explored to improve lattice matching, such as ZnMgSe (used as intermediate shell layers). Houtepen et al. reported that MgSe interlayers, which exhibit near‐perfect lattice matching with InP, can enhance the interface between InP cores and ZnS shells [102]. However, this method resulted in relatively broad emission spectra (50 nm FWHM). Building on this concept, Wang et al. designed an alloyed inner‐shell structure of InP/Zn(Mg)Se/ZnS QDs. The incorporation of Mg^2+^ into the ZnSe lattice not only can effectively mitigate the lattice mismatch between the core and shell to reduce the emission of surface defect states, but also can increase the band gap of Zn(Mg)Se to confine the exciton delocalization from core to shell. This optimized structure achieved remarkable performance with a narrow FWHM (36 nm) and a high PL QY (88%) [103].

To address the substantial lattice mismatch between InP cores (5.87 Å) and ZnS shells (5.41 Å), researchers typically incorporate gradient ZnSeS or Zn(Mg)Se intermediate shells. While this approach reduces strain, it introduces new interfacial defects. Recent studies demonstrate that controlled introduction of metal ions can substantially improve the properties of QDs. For instance, Jiang et al. achieved high‐performance QDs through dual‐stage addition of aluminum isopropoxide (AIP) during ZnSeS/ZnS shell growth, realizing QDs with 96% PL QY and 37 nm FWHM (Figure 8D) [58]. Their proposed mechanism suggests aluminum species simultaneously mitigate core/shell charge mismatch, passivate interfacial defects, and form protective surface oxides that enhance environmental stability. Similarly, Liu et al. reported that treating the green‐emitting InP core with Al, Ga, or In salts before ZnSeS inner shell growth can effectively passivate phosphorus dangling bonds on the core surface. Furthermore, a small amount of Mn ions doping in the second step of ZnSeS inner‐shell growth was found to modify the crystal structure of ZnSeS, thereby reducing interfacial defects and strain. Through surface metal salt modulation and inner‐shell Mn doping, the best performance was achieved with Ga‐InP/Zn(Mn)SeS/ZnS QDs, showing an FWHM of 35 nm and a PL QY of 94% [104]. The incorporation of specific metal ions during shell growth and core pretreatment offers a promising pathway to simultaneously address lattice mismatch, reduce interfacial defects, and enhance the optical performance and stability of InP‐based QDs.

### 3.4 Engineering of Surface Ligand

Surface chemistry modification serves as an effective strategy for enhancing the photophysical properties of QDs. First, surface ligands can passivate surface defects, thereby improving the stability of QDs [105]. Second, the ligand length is one of the key factors affecting carrier mobility [106]. Furthermore, variations in the chemical binding groups and dipole moments of ligands can alter the surface dipole strength, leading to shifts in the valence band maximum (VBM) and conduction band minimum (CBM) of QDs [107, 108]. Therefore, surface engineering plays an essential role in the surface properties, stability, and optoelectronic performance of QDs.

**FIGURE 9:**
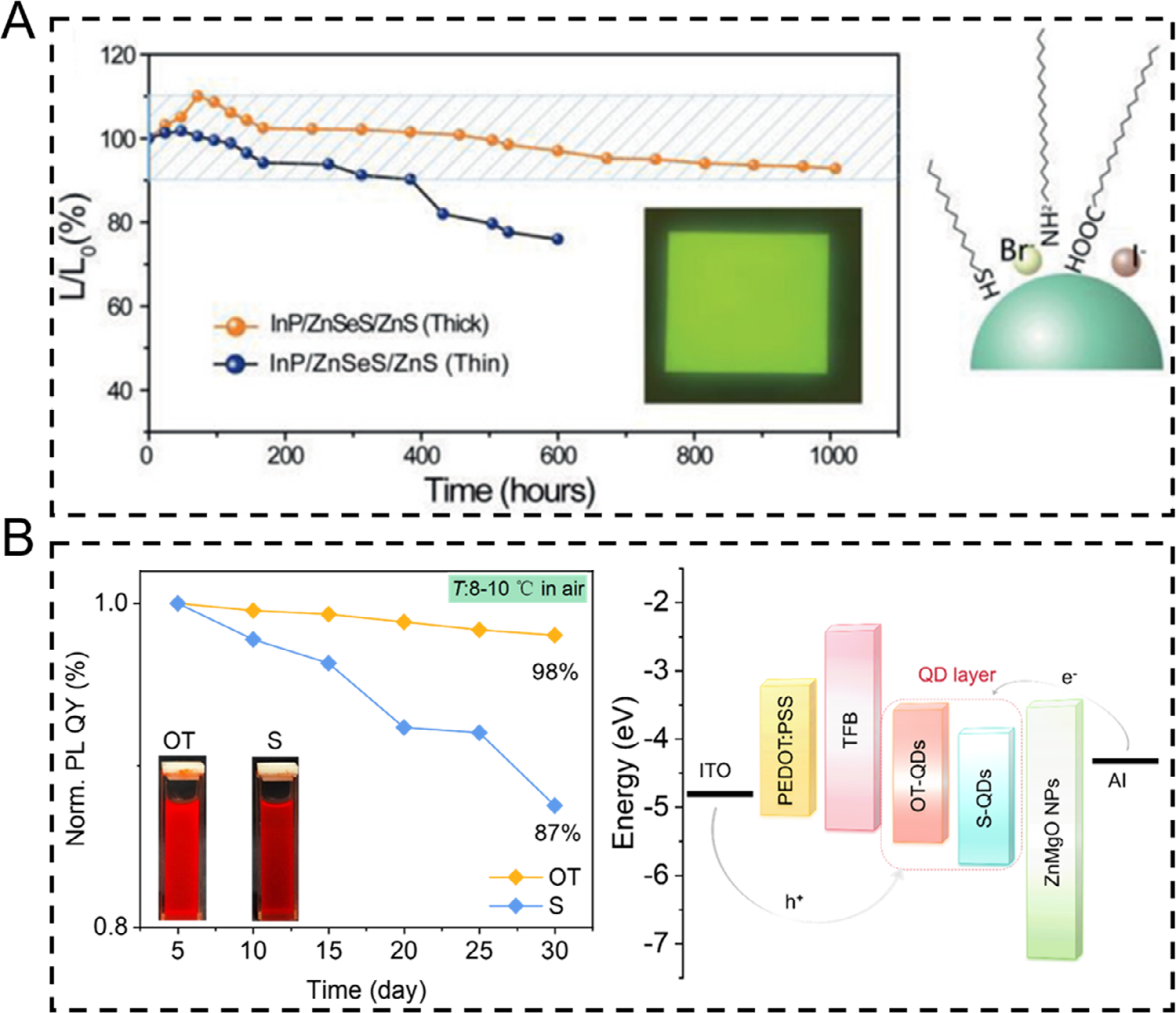
(A) The stability test of QD with thick shells and thin shells. Reproduced with permission [75]. Copyright 2021, John Wiley & Sons. (B) Stability of OT and S‐based QDs in air environment (left), and schematic energy‐level diagram of multilayered QLEDs (right). Reproduced with permission [115]. Copyright 2025, John Wiley & Sons.

InP QDs synthesized via aminophosphine precursors typically employ oleylamine as both solvent and ligand, with InX_3_ (X = Cl, Br, I) as the indium source and ZnX_2_ as a stabilizing agent. This synthetic approach typically yields QDs with mixed surface ligands comprising oleylamine and halide ions. However, the effectiveness of surface passivation highly depends on the precise matching between ligand and surface defect states. As mentioned, L‐type ligands, such as oleylamine, saturate the coordinatively unsaturated sites of surface metal cations through coordinate bonds, primarily providing colloidal stability and basic electronic state modification, but their ability to passivate anionic vacancies (e.g., phosphorus vacancy) is limited [109]. In contrast, X‐type ligands, such as halide ions and thiols, form stronger ionic or covalent bonds with surface metal ions, more effectively filling metal cation vacancies (e.g., phosphorus vacancy, indium vacancy, and zinc vacancy), thereby suppressing the formation of mid‐gap states that act as non‐radiative recombination centers [70, 110]. For instance, Sun et al. revealed that surface‐bound halogen atoms could prevent oxidation, enabling InP QD‐based color enhancement films to achieve exceptional operational stability exceeding 1,000 h (Figure 9A) [75].

Furthermore, different passivation strategies profoundly influence exciton dynamics by modulating the energy distribution and density of surface states, thereby affecting both non‐radiative Auger recombination and radiative recombination pathways [23, 111, 112, 113, 114]. Strongly bound X‐type ligands (e.g., short‐chain thiols) can effectively reduce surface defect state density, significantly suppressing Auger recombination and improving PL QY. However, overly long insulating ligand chains (e.g., long‐chain alkyl amines/thiols) may introduce excessively high carrier injection barriers, which is detrimental to charge transport in QLEDs. This is exemplified by Sun et al.’s work, in which replacing 1‐dodecanethiol with 1‐octanethiol improved carrier injection efficiency, yielding blue InP‐based QLEDs with a record EQE of 2.6% [113]. Additionally, our group further confirmed the effectiveness of 1‐octanethiol ligand in improving QD stability and promoting radiative recombination in the device. The 1‐octanethiol‐optimized QDs exhibited exceptional stability, retaining 98% of their initial PL QY after 1 month in ambient air. Moreover, the 1‐octanethiol ligand could raise the energy level of the emitting layer, reducing the hole injection barrier and enhancing radiative recombination (Figure 9B). The resulting QLEDs achieved a record EQE of 21.55% and a maximum luminance of 95,327 cd m^−2^. Remarkably, the device also demonstrated a long operational lifetime, with a *T*_50_ exceeding 50,000 h, setting a new state‐of‐the‐art performance for (DMA)_3_P‐based QLEDs [115].

Therefore, a balance must be struck between “passivation efficiency” and “charge injection efficiency”. Advanced passivation strategies are moving towards developing “locked” ligands, such as using bidentate or multidentate ligands (e.g., dithiols and phosphonic acids) to enhance binding stability, or constructing ultrathin inorganic shells (e.g., ZnO and Al_2_O_3_) on the QD surface via atomic layer deposition (ALD) to achieve dual protection through physical isolation and chemical passivation.

## 4 Advances Achieved for QLEDs With (DMA)_3_P‐Based InP QDs

### 4.1 Mechanisms of Degradation for InP‐Based QLEDs

In typical QLEDs, EQE is a crucial parameter to evaluate their performance. According to the equation EQE = *ƞ*_rad_*ƞ_rec_*ƞ_ext_ (where *ƞ*_rad_ is the PL QY, *ƞ*_rec_ is the recombination rate, *ƞ*_ext_ is the light extraction rate (or light coupling)), the PL QYs, the charge injection balance in the QD emitter layers and light extraction rate are critical factors determining device performance. To achieve efficient PL QYs, key parameters, such as core, core/shell structure, and surface ligand must be optimized, as detailed in Section 3.

From a device perspective, charge injection balance is not only crucial for efficiency but also a major factor limiting operational lifetime. This is because unbalanced charge injection can trigger undesirable effects, notably charge accumulation and leakage currents. At present, the most common device architecture for InP‐based QLEDs is adopted from Cd‐based QLED designs. However, InP‐based QDs generally exhibit a shallower VBM compared to Cd‐based QDs, which facilitates hole injection but hinders electron injection. Additionally, the small energy barrier between the QDs and the hole transport layer (HTL) can allow injected electrons to leak into the HTL. These accumulated carriers can break chemical bonds (such as C‐N in the HTL), leading to the HTL degradation, and can also react with the surface ligands of QDs, causing ligand dissociation and inducing numerous surface defects [116, 117, 118]. Furthermore, unbalanced charge injection can cause the generation of Joule heat in the emitting layer, which can cause significant efficiency roll‐off at high voltages and shorten device lifetime [119]. Therefore, both optimizing QD luminescent materials and engineering device structures are essential for enhancing device performance. In the following section, we will focus on strategies to improve performance through device structure optimization.

### 4.2 Development of InP‐Based QLEDs

**FIGURE 10:**
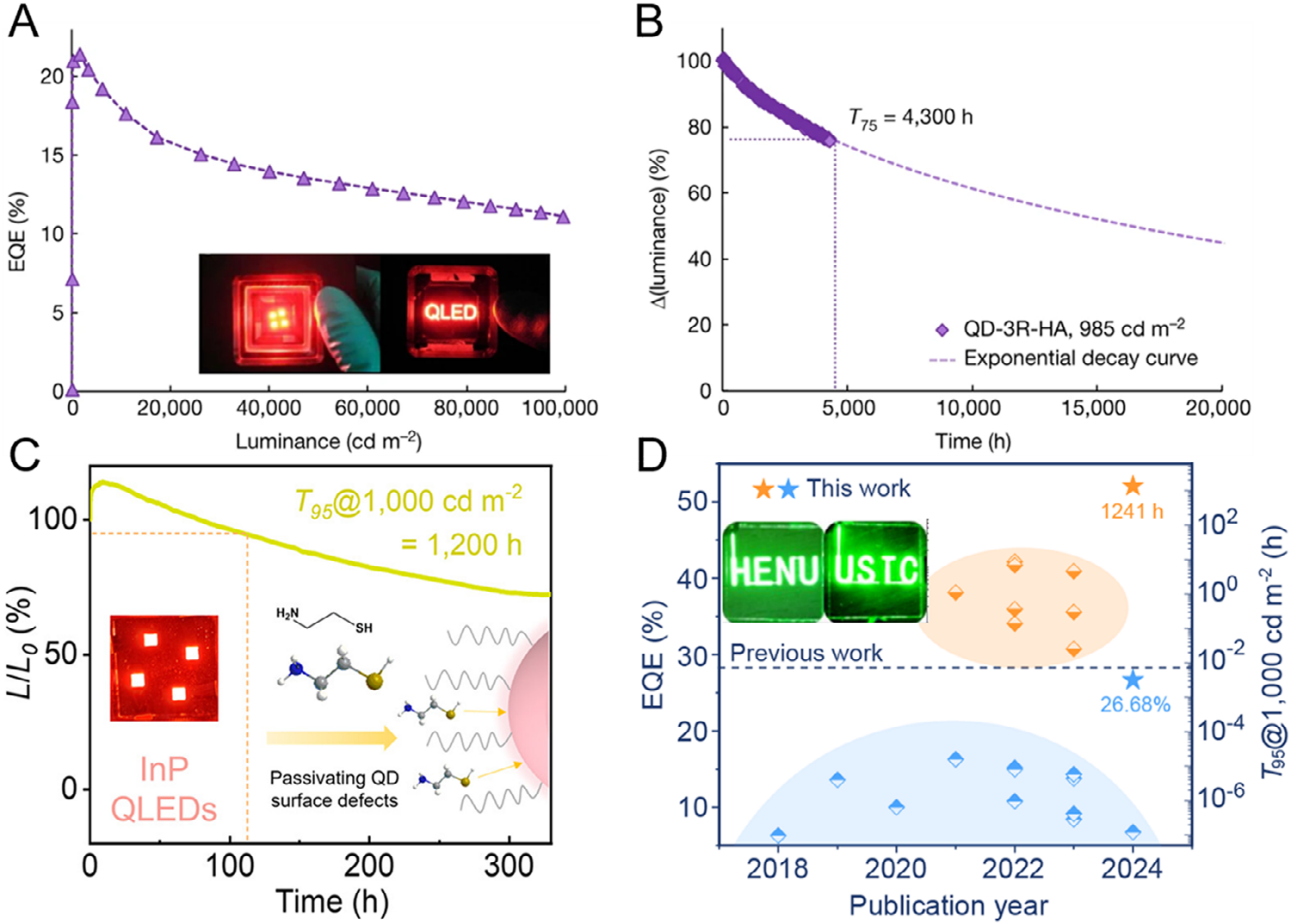
(A) EQE‐luminance characteristics and (B) lifetime measurement of the QLEDs with QD‐3R‐HA. Reproduced with permission [23]. Copyright 2019, Springer. (C) Lifetime measurement of the QLEDs with CTA. Reproduced with permission [121]. Copyright 2024, American Chemical Society. (D) Comparison of EQE and *T*_95_ lifetime with previously reported values. Reproduced with permission [24]. Copyright 2022, American Chemical Society.

As a leading cadmium‐free QD material, InP‐based QDs exhibit precisely tunable emission across the visible to near‐infrared spectrum through controlled size adjustment, demonstrating exceptional color purity with narrow spectral bandwidth. These superior optical characteristics make InP‐based QDs ideally suited for next‐generation wide‐color‐gamut QLED displays, simultaneously addressing the stringent performance demands and environmental compliance requirements of premium consumer electronics, including ultra‐high‐definition televisions and mobile displays. However, the development of InP‐based QLEDs has lagged significantly behind their Cd‐based counterparts. It was not until 2011 that the first InP‐based QLEDs using InP@ZnSeS QDs were reported, although their device efficiency remained below 0.1%. These early limitations were primarily attributed to underdeveloped synthesis techniques at the time, yielding QDs with poor PL QYs below 50% [120]. However, recent years have seen remarkable progress through multifaceted optimizations, including synthetic methodology innovations, in‐situ core etching techniques, core/shell structure engineering, surface ligand modifications, and device architecture refinements. Red‐emitting InP‐based QLEDs have emerged as the frontrunners in this technological race. A pivotal advancement came in 2019 when Peng's group achieved near‐perfect red‐emitting InP/ZnSe/ZnS core/shell QDs through precise stoichiometric control during nucleation and shell growth. These QDs exhibited exceptional characteristics with near‐unity PL QY (∼100%), a narrow FWHM of 42 nm, monoexponential decay dynamics, and nonblinking behavior, enabling QLEDs with an EQE of 12.2% and a maximum luminance exceeding 10,000 cd m^−2^ [68]. Simultaneously, by employing in‐situ HF core etching and growing a ZnSe shell under high temperature, Won's team achieved record‐breaking red QLEDs with an EQE of 21.4%, a maximum luminance surpassing 100,000 cd m^−2^, and unprecedented operational stability (*T*_95_ = 615 h at 1,000 cd m^−2^) (Figure 10A,B) [23]. The performance ceiling was further elevated in 2025 by Shen's introduction of bifunctional cetyltrimethylammonium (CTA) ligands, which simultaneously passivated surface defects and created interfacial dipoles for balanced charge injection. Consequently, they achieved red InP‐based QLEDs with a *T*_95_@1,000 cd m^−2^ exceeding 1,200 h (Figure 10C) [121]. On the other hand, green InP QLEDs recently achieved an EQE of 26.6%, a luminance exceeding 270,000 cd m^−2^, and a *T*_95_@1,000 cd m^−2^ of over 1,200 h, establishing new benchmarks for green InP‐based QLED performance (Figure 10D) [24].

However, most of the above high‐performance InP QDs were synthesized using (TMS)_3_P as the phosphorus source. As shown in Table 2, compared with (TMS)_3_P‐based QLEDs, the development of (DMA)_3_P‐based QLEDs is relative slow, which may be attributed to the following: (1) the difficulty to synthesize (DMA)_3_P‐based QDs with high PL QY and uniform size; (2) the severe efficiency roll‐off under high voltage due to the instability of surface ligands on (DMA)_3_P‐based QDs; (3) the imbalanced charge injection owing to the different energy structure between (TMS)_3_P‐based QDs and (DMA)_3_P‐based QDs.

**TABLE 2:** Summary of PL and EL parameters for (TMS)_3_P‐based and (DMA)_3_P‐based QDs and QLEDs.

(TMS)_3_P									
	Year	PL (nm)/ PL QY (%)	Device structure	V_on_ (V)	EQE (%)	CE (cd A^−1^)	Luminance (cd m^−2^)	Operational lifetime (h)	Ref.
Red	2016	607/82	ITO/PEDOT:PSS/TFB/InP/ZnSeS/ZnS QDs/ZnO/Al	3.0	2.5	4.2	2,849	—	[122]
	2018	607/73	ITO/ZnO/InP/ZnSe/ZnS QDs/CBP/HATCN/Al	2.0	6.6	13.6	1,600	—	[123]
	2019	618/93	ITO/PEDOT:PSS/poly‐TPD/InP/ZnSe/ZnS QDs/ZnMgO/Ag	1.8	12.2	14.7	>10,000	—	[68]
	2019	630/∼100	ITO/PEDOT:PSS/TFB/ InP/ZnSe/ZnS QDs/ZnMgO/Al	1.8‐2.0	21.4	—	100,000	*T*_50_ = 1,000,000 h @ 100 cd m^−2^	[23]
	2020	632/92	ITO/ZnMgO/QDs/TCTA/PCzAC/DBTA/PCBBiF/HATCN/Al	3.5	21.8	23.4	23,300	*T*_50_ = 1,095 h @ 1,000 cd m^−2^	[124]
	2020	616/81.8	ITO/PEDOT:PSS/F4‐TCNQ/TFB/ InP/ZnSeS/ZnS QDs/Cl@ZnMgO/Al	2.0	4.0	6.3	1,977	—	[57]
	2021	630/‐	ITO/PEDOT:PSS/TFB/InP/ZnSe/ZnS QDs/ZnMgO@MPTES/ZnMgO/Al	1.6	14.70	19.20	3,301	*T*_50_>550 h @1,000 cd m^−2^	[125]
	2021	630/95	ITO/PEDOT:PSS/TFB/TPD: InP/ZnSe/ZnS QD/ZnMgO/Al	—	18.6	—	128,577	*T*_70_ = 107,772 h @ 100 cd m^−2^	[126]
	2022	620/>90	ITO/PEDOT:PSS/TFB/InP/ZnSe/ZnS QDs/ZnMgO/Al	1.87	22.2	—	>110,000	*T*_95_>32,000 h @ 100 cd m^−2^	[78]
	2022	623/92	ITO/PEDOT:PSS/TFB/InP/ZnSe/ZnS QDs/ZnMgO/Al	1.8	22.56	26.70	107,160	*T*_50_ = 110,702 h @ 100 cd m^−2^	[70]
	2022	630/90	ITO/PEDOT:PSS/NiOx/Poly‐TPD/InP/ZnSe/ZnS QDs/ZnMgO/Al	2.0	18.8	—	22,300	*T*_75_ = 74 h @ 2,500 cd m^−2^	[127]
	2022	630/86	ITO/ZnO/InP/ZnSe/ZnS QD/DBT/PCBBi/HATCN/Al	2.0	10.6	10.4	>8,000	*T*_50_ = 164,048 h @ 100 cd m^−2^	[128]
	2023	625/80	ITO/PEDOT:PSS/B‐PTAA/ InP/ZnS QDs/ZnMgO/Ag	2.0	20.4	25.3	24,000	—	[129]
	2024	614/>90	ITO/PEDOT:PSS/PF8Cz/InP/ZnSe/ZnS QDs/ZnMgO/Al	1.9	21.21	27.84	124,220	*T*_95_ = 1,200 h @ 1,000 cd m^−2^	[121]
	2025	614/∼92	ITO/PEDOT:PSS/PF8Cz/InP/ZnSe/ZnS QDs/PVP/ZnMgO/Al	2.0	23.5	37.70	—	*T*_95_ = 835 h @ 1,000 cd m^−2^	[130]
	2025	622/83	ITO/PEDOT:PSS/TFB/QDs/ZnMgO/Al	2.0	26.6	—	140,000	*T*_50_ = 4,026 h @ 1,000 cd m^−2^	[131]
Green	2011	522/>50	ITO/PEDOT:PSS/poly‐TPD/InP/ZnSeS QDs/TPBi/LiF/Al	—	0.008	—	—	—	[120]
	2012	550/55	ITO/PEDOT:PSS/poly‐TPD/InP/ZnSeS QDs/TPBi/LiF/Al	4.5	0.26	—	700	—	[132]
	2013	500/72	ITO/ZnO/PFN/InP/ZnSeS QDs/TCTA/MoO_3_/Al	2.2	3.46	10.9	3,900	—	[133]
	2015	507/44	ITO/PEDOT:PSS/poly‐TPD/InP/ZnSe/ZnS QDs/TPBi/Ca or Ag	3.7	—	1.5	448	—	[134]
	2017	525/70	ITO/ZnMgO/InP/ZnSeS/ZnS QDs/TCTA/NPB/HATCN/Al	2.2	∼1.5	4.44	10,490	—	[135]
	2019	527/70	ITO/PEDOT:PSS/TFB/InP/GaP/ZnS/ZnS QDs/ZnO/Al	—	6.3	13.7	2,938	—	[136]
	2019	531/82	ITO/PEDOT:PSS/poly‐TPD/PVK/QDs/ZnMgO/ZnO/Al	—	13.6	—	13,900	—	[137]
	2021	535/86	ITO/ZnMgO/InP/ZnSe/ZnSQDs/TCTA/MoO_3_/Al	2.2	16.3	57.5	12,646	*T*_50_ = 1,033.4 h @ 100 cd m^−2^	[110]
	2022	‐/91	ITO/PEDOT:PSS/TFB/PVP/InP/ZnSe/ZnS QDs/ZnO/Al	—	10.6	40.7	15,606	*T*_50_ = 5,462 h @ 100 cd m^−2^	[138]
	2022	526/90	ITO/PEDOT:PSS/TFB/InP/ZnSe/ZnS QDs/ZnMgO/Al	2.2	7.8	25.8	4,955	*T*_50_ = 402 h @ 100 cd m^−2^	[139]
	2022	528/89	ITO/ZnO/ZnS/In(Zn)P/ZnSeS/ZnS QD/DBTA/PCBBiF/HATCN/Al	2.4	10.8	37.5	1,756	*T*_50_ = 60,255 h @ 100 cd m^−2^	[140]
	2023	532/90	ITO/PEDOT:PSS/PTAA/InP/ZnSe/ZnS QDs/ZnMgO/Al	2.2	13.8	52.2	16,788	*T*_50_ = 5,944 h @ 100 cd m^−2^	[141]
	2024	532/79	ITO/s‐FZO/InZnP/ZnSeS/ZnS QDs/DBTA/PCBBiF/HAT‐CN/Al	2.35	20.07	68.92	8,635	*T*_50_ = 542 h @ 1,500 cd m^−2^	[142]
	2024	534/91	ITO/PEDOT:PSS/PF8Cz/QDs/ZnMgO/Al	2.0	12.74	54.56	175,000	*T*_50_ = 20,044 h @ 100 cd m^−2^	[143]
	2024	540/95	ITO/PEDOT:PSS/PF8Cz/InP/ZnSe/ZnS QDs/ZnMgO/Al	2.1	26.68	112.56	277,000	*T*_95_ = 1,241 h @ 1,000 cd m^−2^	[24]
	2025	538/96	ITO/PEDOT:PSS/PF8Cz/InP/ZnSeS/ZnS QDs/ZnMgO/Al	—	21.43	90.45	255,985	*T*_50_ = 290,000 h @ 100 cd m^−2^	[144]
	2025	∼530/>90	ITO/PEDOT:PSS/PF8Cz/InP/ZnSeS/ZnS QDs/ZnMgO/Al	2.0	26.3	108.3	—	—	[145]
Blue	2020	480/81	ITO/PEDOT:PSS/TFB/InP/GaP/ZnS/ZnS QDs/ZnO/Al	—	1.01	—	3,120	*T*_50_ = 2 h @ 100 cd m^−2^	[146]
	2020	485/45	ITO/PEDOT:PSS/PVK:poly‐TPD/InP/GaP/ZnS QDs/ZnO/Al	2.0	1.0	3.6	1,045	—	[111]
	2020	486/65	ITO/ZnO/PFN/InGaP@ZnS QDs/TCTA/MoO_3_/Al	5.0	0.20	—	74	—	[147]
	2022	482/‐	ITO/PEDOT:PSS/MoO_3_/TFB/InP/ZnS QDs/ZnMgO/Al	—	2.82	—	421	—	[148]
	2025	474/73	ITO/PMA/TFB/InP/ZnS QDs/ZnMgO/Al	2.75	2.38	—	2,693	*T*_50_ = 228 h @ 100 cd m^−2^	[53]
	2025	468/75	ITO/PEDOT:PSS/PF8Cz/InP/ZnS QD/ZnMgO/Al	—	4.1	7.2	624	—	[149]
(DMA)_3_P									
Red	2022	617/85	ITO/PEDOT:PSS/TFB/InP/ZnSe/ZnS QDs/ZnMgO/Al	—	12.0	14.1	16,954	*T*_50_ = 114.6 h @ 250 cd m^−2^	[150]
	2022	620/90	ITO/PEDOT:PSS/TFB/InP/ZnSe/ZnS QDs/ZnMgO/TPBi/LiF/Al	2.0	5.07	5.4	21,070	*T*_50_ = 378 h @ 100 cd m^−2^	[151]
	2022	621/86	ITO/PEDOT:PSS/TFB/InP/ZnSe/ZnS QDs/ZnMgO/Al	—	8.9	8.8	13,395	—	[76]
	2023	635/83.5	ITO/PEDOT:PSS/TFB/InP/ZnSe/ZnS QDs/ZnMgO/Al	2.6	8.1	11.1	17,759	*T*_50_ = 14.68 h @ 100 cd m^−2^	[152]
	2023	618/96	ITO/PEDOT:PSS/TFB/InP/ZnSe/ZnSeS/ZnS QDs/ZnMgO/Al	2.5	12.39	19.76	44,160	*T*_50_ = 13,660 h @ 100 cd m^−2^	[89]
	2023	680/95	ITO/PEDOT:PSS/TFB/InP/ZnSe/ZnSeS/ZnS QDs/ZnO/Al	1.8	6.5	—	2,263	—	[153]
	2023	625/85	ITO/ZnO/CBP‐InP/ZnSe QDs/PVK/WO_3_/Au	1.8	11.6	—	14,600	—	[154]
	2025	602/93	ITO/PEDOT:PSS/TFB/InP/ZnSe/ZnSeS/ZnS QDs/ZnMgO/Al	—	11.8	—	6,325	—	[36]
	2025	615/96	ITO/PEDOT:PSS/TFB/ InP/ZnSe/ZnSeS/ZnS QDs/ZnMgO/Al	1.9‐ 2.0	21.55	30.08	95,327	*T*_50_ = 50,066 h @ 100 cd m^−2^	[115]
Green	2017	530/60.1	ITO/HIL/HTL/InP/ZnS QDs/EBL/ETL/EIL/Al	∼5	0.223	0.65	160	—	[155]
	2020	537/82	ITO/PEDOT:PSS/TFB/ InP/ZnSeS QDs/ZnO/Al	—	0.904	2.98	1,593	—	[156]
	2021	518/67.5	ITO/PEDOT:PSS/TFB/ InP/ZnSeS/ZnS QDs/ZnMgO/Al	2.5	1.68	4.79	725	—	[157]
	2021	517/95	ITO/ZnMgO/InP/ZnSeS/ZnSQDs/TCTA/MoO_3_/Al	2.9	7.06	—	1836	—	[75]
	2022	528/97	ITO/PEDOT:PSS/TFB/InP/ZnSeS/ZnS QDs/ZnMgO/Al	—	15.2	—	2300	—	[35]
	2022	531/87	ITO/PEDOT:PSS/TFB/InP/ZnSe/ZnS QDs/ZnMgO/Al	7.0	9.3	36.6	13,445	—	[150]
	2023	526/91	ITO/PEDOT:PSS/Poly‐TPD/InP/ZnSeS/ZnS QDs/LiF/ZnMgO/Al	—	5.56	—	2,798	—	[101]
	2025	525/93	ITO/PEDOT:PSS/TFB/InP/ZnSeS/ZnS QDs/ZnMgO/Al	—	4.6	—	13,000	—	[158]
	2025	509/88	ITO/PEDOT:PSS/TFB/InP/ZnSe/ZnSeS/ZnS QDs/ZnMgO/Al	—	7.5	—	3,842	—	[36]
Blue	2020	465/82	ITO/PEDOT:PSS/PVK/InGaP/ZnSeS/ZnS QDs/ZnMgO/Al	—	2.5	3.8	1,038	—	[95]
	2020	468/45	ITO/PEDOT:PSS/PVK/QDs/ZnMgO/Al	—	1.7	—	140	—	[98]
	2021	468/45	ITO/PEDOT:PSS/MoO_3_/PVK/InP/ZnS/ZnS QDs/ZnMgO/Al	—	2.1	—	165	—	[159]
	2022	474/93	ITO/PEDOT:PSS/TFB/InP/ZnS/ZnS QDs/ZnMgO/Al	2.9	2.6	—	422	—	[113]
	2025	452/‐	ITO/PEDOT:PSS/TFB/InP/ZnS/ZnS QDs/ZnMgO/Al	3.0	0.8	—	275	—	[160]

**FIGURE 11:**
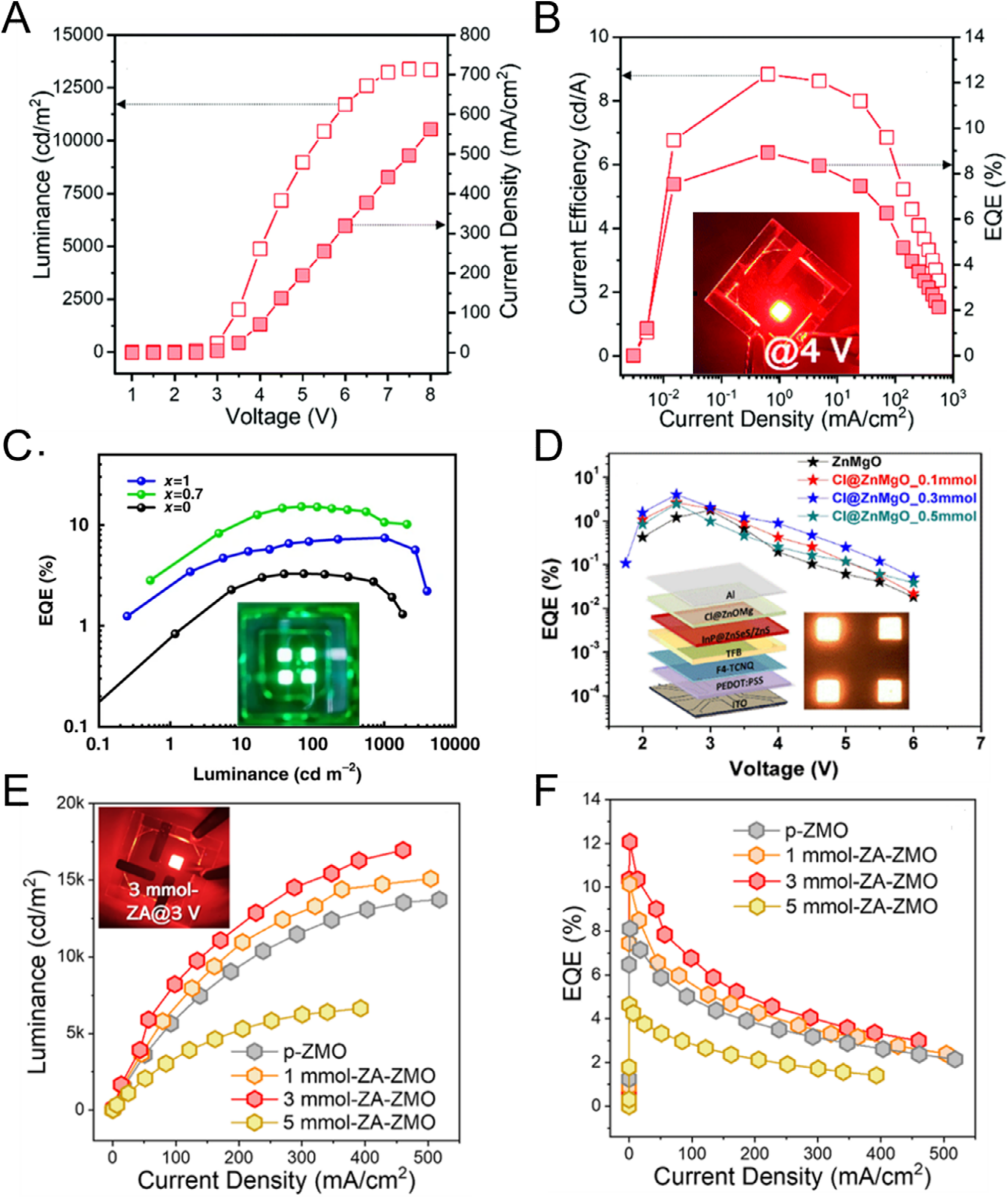
(A) Luminance‐current density‐voltage characteristics and (B) current efficiency‐EQE‐current density characteristics of red QLEDs with InP/thick‐ZnSe/ZnS QDs. Reproduced with permission [76]. Copyright 2022, Royal Society of Chemistry. (C) EQE‐luminance characteristics of green QLEDs with InP/ZnSe_x_S_1‐x_/ZnS QDs. Reproduced with permission [35]. Copyright 2022, Springer. (D) EQE‐voltage characteristics of QLEDs with Cl‐doped ZnMgO NPs. Reproduced with permission [57]. Copyright 2020, American Chemical Society. (E) Luminance‐current density and (F) EQE‐current density characteristics of QLEDs with acrylate‐functionalized ZnMgO NPs. Reproduced with permission [150]. Copyright 2022, American Chemical Society.

To narrow the performance gap between (TMS)_3_P‐based QLEDs and (DMA)_3_P‐based QLEDs, researchers have made improvements in the engineering of QD emitting materials, charge transport materials, and the modification of the emitting layer/charge transport layer interface. For instance, in 2022, Yang et al. developed (DMA)_3_P‐based red InP QDs exhibiting an exceptional PL QY of 86% with a narrow FWHM of 44 nm. This achievement was realized through the innovative use of InBr_3_ as an alternative indium precursor to conventional InCl_3_, coupled with an optimized ZnSe inner shell thickness. When implemented as red electroluminescent emitting materials, these high‐performance QDs demonstrated outstanding device characteristics, achieving a record‐breaking luminance of 13,395 cd m^−2^ and an EQE of 8.9% (Figure 11A,B) [76]. In the same year, Zhao's research team addressed the critical issue of lattice mismatch between ZnSe and ZnS shells by introducing an innovative gradient inner shell layer of ZnSe_x_S_1‐x_. This strategic modification effectively mitigated interface defects through improved lattice matching while simultaneously enabling precise tuning of the energy levels in InP‐based QDs, thereby facilitating more balanced carrier injection. The resulting QLEDs exhibited a maximum EQE of 15.2%, establishing a new performance benchmark for InP‐based pure green‐emitting QLEDs utilizing environmentally friendly (DMA)_3_P as the phosphorus precursor (Figure 11C) [35].

The superior performance of QLEDs is dictated not merely by the exceptional intrinsic properties of the QD emitting material itself, but to a far greater extent by the meticulous engineering of the device architecture. This critical engineering effort encompasses several interdependent factors: (1) Precise energy level alignment across all functional layers is paramount to minimize energy barriers, thereby facilitating the efficient flow of charge carriers and reducing efficiency‐loss mechanisms such as non‐radiative recombination. (2) Efficient charge injection and transport dynamics must be carefully balanced to ensure that electrons and holes are supplied to the QD emitting layer at comparable rates, preventing charge quenching and optimizing the likelihood of radiative recombination. (3) The structural and electronic integrity of interlayer interfaces is essential, as defective interfaces can introduce trapping sites, act as centers for exciton quenching, and ultimately compromise device stability and operational lifetime.

A pivotal study by Yang et al. in 2016 systematically compared two hole transport materials (TFB and PVK) with distinct hole mobilities. Their results showed that TFB‐based devices exhibited blue EL due to the relatively deep LUMO level of TFB (compared to PVK), which could not entirely prevent electron leakage from QDs to TFB. However, owing to TFB's significantly higher hole mobility (∼10^−^^2^ cm^2^ V^−^^1^ s^−^^1^) than PVK (10^−^^6^–10^−^^5^ cm^2^ V^−^^1^ s^−^^1^), TFB‐based devices achieved higher luminance and efficiency [122]. Thus, improving hole injection while suppressing electron leakage is essential for enhancing InP‐based QLED performance. Building on this understanding, Kim et al. implemented an innovative approach using F4‐TCNQ as a p‐type dopant at the Poly(3,4‐ethylenedioxythiophene):poly‐(styrenesulfonate) (PEDOT:PSS)/TFB interface. This strategic modification effectively lowered hole injection barriers, yielding a notable EQE enhancement to 3.87% [161]. PEDOT:PSS is widely used as the HTL in QLEDs. It provides a smooth surface for subsequent layers and exhibits a favorable work function, enabling efficient hole injection from the indium tin oxide (ITO) anode into the HTL. However, its inherent limitations, particularly the acidic nature and poor ionic conductivity of the PSS component, contribute to device degradation and suboptimal charge transport. Chae et al. addressed this fundamental limitation through a breakthrough two‐step solvent treatment (ethylene glycol (EG) and methanol (MeOH)), which effectively reduces PSS content by 40%, promoting a conformational transition of PEDOT from benzoid to quinoid structure. These structural modifications dramatically improved electrical conductivity (50 → 1151 S/cm) and hole transport characteristics, ultimately doubling the operational lifetime (51.2 → 125.6 h) while boosting device efficiency to 6.4% [162].

Zinc oxide nanoparticles (ZnO NPs) have become the most widely adopted electron transport layer (ETL) material in QLEDs due to their outstanding electron mobility, efficient hole‐blocking capability, and optimal energy band alignment with QDs [137, 150, 163, 164, 165]. However, ZnO NPs typically exhibit numerous lattice imperfections and surface defects that act as charge‐trapping centers, resulting in substantial exciton quenching at the interface between the QD emissive layer (EML) and ETL. Extensive research has been conducted to address the issues of unbalanced charge injection and interfacial exciton quenching associated with ZnO NP‐based ETLs [57, 141, 166, 167]. For example, Chea et al. achieved balanced carrier injection by adjusting the Mg ratio in ZnMgO, realizing green InP‐based QLEDs with an EQE of 1.68% [141]. Furthermore, by incorporating Cl doping into ZnMgO, the same research group significantly reduced the injection barrier at the QD/ETL interface, further improving charge balance and enhancing the EQE of red InP‐based QLEDs from 1.8% to 4% (Figure 11D) [57]. In addition, to suppress exciton quenching at the EML/ETL interface, Yang et al. modified the surface of ZnMgO NPs with acrylate functional groups. This modification not only alleviated emission quenching at the interface but also elevated the energy levels of the ETL, promoting better charge balance. The optimized device demonstrated significantly improved EL performance, achieving a luminance of 16,954 cd m^−^^2^ and an EQE of 12.0% (Figure 11E,F). The applicability of acrylate‐functionalized NPs was further validated in green InP QLEDs, which attained a luminance of 13,445 cd m^−^^2^ and an EQE of 9.3% [150].

In contrast to their red and green counterparts, blue (DMA)_3_P‐based QLEDs demonstrate inferior electroluminescent performance, attributed to the increased bandgap. (TMS)_3_P exhibits high reactivity and fast reaction kinetics, making them challenging to synthesize small‐sized blue‐emitting InP cores [109, 146]. To overcome this challenge, researchers have turned to amino‐phosphines as an alternative phosphine source. Using (DMA)_3_P, Yang et al. successfully prepared blue‐emitting InGaP/ZnSeS/ZnS QDs via an In^3+^‐to‐Ga^3+^ cation‐exchange strategy, achieving devices with a maximum luminance of 1,038 cd m^−2^ and an EQE of 2.5% [96]. Subsequently, Sun et al. further improved the EQE to 2.6% by replacing long‐chain 1‐dodecanethiol with short‐chain 1‐octanethiol [111].

Overall, the device performance based on (DMA)_3_P has been improved through the engineering of QD emitting materials, the charge transport material, and the interface between the emitting layer and charge transport layer. However, there is still significant room for improvement in efficiency, luminance, and operational lifetime compared to devices based on (TMS)_3_P.

## 5 Challenges and Perspectives

Despite significant advancements in the performance of InP‐based QDs and their corresponding QLEDs, they still exhibit considerably inferior performance compared to Cd‐based counterparts and remain below the threshold for practical commercialization. To bridge this gap, further optimization across the entire development chain—from material synthesis to device engineering—remains highly valuable and warrants continued exploration to accelerate the commercialization of InP QDs. Here, we have summarized current research challenges and potential future directions in this field:

1. Unresolved mechanisms in nucleation dynamics

Although prior studies have confirmed the feasibility of synthesizing InP cores using (DMA)_3_P as the phosphorus precursor, key aspects of the process remain unresolved. Specifically, the low‐temperature (<300°C) decomposition pathway of (DMA)_3_P, as well as the structure, stability, and transformation mechanism of transient In‐P coordination intermediates during early‐stage nucleation, lack direct in‐situ experimental validation. Moreover, the roles of solvent coordination strength (e.g., octadecene and oleylamine), reaction temperature, and (DMA)_3_P/In molar ratio in governing nucleation kinetics and size dispersity are not yet systematically established, hindering precise control over monodisperse InP core formation. To address these gaps, an integrated approach—combining in‐situ spectroscopy (Fourier transform infrared spectroscopy (FTIR), Raman), synchrotron X‐ray absorption spectroscopy (XAS), and DFT‐assisted molecular dynamics (DFT‐MD) simulations—should be proposed to unravel the atomistic dynamics of (DMA)_3_P decomposition and InP nucleation. Concurrently, advancing surface ligand engineering strategies will enhance nucleation uniformity, thereby establishing a robust framework for the controlled synthesis of high‐quality InP‐based QDs.

2. Challenges in fabricating large‐sized (>20 nm) InP core/shell QDs

A previous study indicates that the number of luminescent centers per unit area in the emitting layer significantly influences charge carrier transport pathways and recombination rates/efficiency. Through systematic optimization of QD size and film thickness, our research team has established that a monolayer of 20 nm QDs in the emitting layer enables superior charge distribution—each QD accommodates a greater average number of charges at equivalent injection levels. This configuration promotes pronounced quasi‐Fermi level splitting and achieves exceptional luminance at reduced operating voltages [9]. However, current synthetic methodologies face fundamental limitations in producing InP core/shell QDs exceeding 10 nm in diameter. These constraints stem from the difficulty in synthesizing large‐sized InP cores as well as the exacerbated lattice strain and associated defect formation resulting from core/shell mismatch at increased dimensions. To overcome these synthetic barriers, a seed‐mediated growth method combined with a temperature‐gradient shell growth strategy, alongside the development of novel ligands and advanced characterization techniques would be proposed. This multifaceted strategy will elucidate the growth mechanisms governing large‐sized InP‐based QD formation, ultimately establishing a robust platform for next‐generation QD optoelectronics.

3. Poor stability of QDs

Experimental observations revealed that InP‐based QDs synthesized using (DMA)_3_P as the phosphorus precursor undergo a rapid decrease in PL intensity during purification and storage. This degradation likely stems from ligand desorption (e.g., oleylamine) from the QD surface over time, which increases surface defect states and enhances non‐radiative recombination, ultimately leading to photoluminescence quenching [119, 167]. Furthermore, oxygen and moisture infiltration may trigger surface oxidation, further accelerating QD degradation. To address this issue, growing a more uniform and thicker shell could enhance the intrinsic structural stability of core/shell QDs. Alternatively, a dual‐ligand strategy (e.g., thioalcohol + amine) or cross‐linking ligands could be employed to improve the binding stability between the ligands and the QD surface.

4. The ambiguous degradation mechanisms of devices hinder substantial advancements in their efficiency, luminance, and operational lifetime.

The performance degradation of InP‐based devices primarily stems from the coupling of multiple physical mechanisms, including material defects, interface states, thermal effects, and non‐radiative carrier recombination [119, 167, 168, 169]. However, the nanoscale dynamics governing these processes and their quantitative contributions remain incompletely understood, resulting in empirically driven rather than mechanism‐guided optimization approaches. Current research is gradually uncovering the atomic‐level origins of degradation through in‐situ characterization techniques and first‐principles calculations. Nevertheless, achieving fundamental breakthroughs still requires the establishment of multi‐scale degradation kinetic models and the development of novel interface regulation and thermal management strategies. In the future, the integration of AI‐driven material screening and in‐situ monitoring technologies may provide new pathways for achieving high efficiency and long lifetime in InP‐based devices.

5. Challenges of InP‐based QD industrial‐scale production

While significant advancements have been achieved in improving the efficiency, luminance, and operational lifetime of InP‐based QLEDs, there are still a few challenges that remain on the path toward their commercialization. Firstly, the large‐scale preparation of high‐color‐purity InP‐based QDs is challenging, as it requires fine control of the entire growth process. Recent advances in AI‐driven material synthesis may offer a promising solution for large‐scale industrial production. Secondly, the maximum efficiencies and luminances are typically achieved after a “positive aging” process, which poses challenges for the mass production of QLEDs. This is because the “positive aging” is usually coincident with the “negative aging” process, which can impede precise color rendering in large‐scale industrial displays [170, 171, 172]. Previous studies suggest that “positive aging” may result from the reactions between ZnO nanoparticles and metal electrodes or between metal electrodes and acidic resin within the device [173, 174, 175]. Hence, adopting doped ZnO ETL with lower reactivity, inserting a buffer layer at the ETL/electrode interface to suppress chemical reactions or ion migration, or avoiding acidic resins may be an effective solution [176]. Thirdly, the low‐cost, high‐volume QLED preparation process is also a challenge. In the laboratory, the spin‐coating technology is commonly employed to improve the film quality of QDs and other solution‐processable functional layers. However, this technique cannot achieve uniform film thickness over large areas, limiting its scalability [177]. Inkjet‐printing has emerged as the most promising method for the mass‐production of QLEDs [178, 179, 180]. Yet, unlike spin‐coating, the absence of centrifugal force in inkjet printing results in lower film quality, leading to inferior EQE and operational lifetime [152, 181]. Thus, to achieve a high‐performance and highly stable active matrix display, the following conditions should be considered, including the selection of the backplane, the design of the pixel‐defining layer, the cavity structure design for Red/Green/Blue full colors, and the subpixel shape and arrangement. With concerted efforts across the field, the future of InP‐based QDs and QLEDs appears exceptionally prosperous.

## 6 Conclusion

In summary, this review comprehensively examined the molecular structures of (TMS)_3_P and (DMA)_3_P, along with their respective reaction mechanisms in InP core nucleation. Additionally, we discussed the key challenges associated with high‐quality InP core. To address problems of high‐density defects, difficulty of carrier injection and ease of carrier leakage, we systematically summarized effective strategies for enhancing the optical and electronic properties of the InP core, including core modulation, core/shell structure design, and surface ligand optimization. Furthermore, we reviewed recent advances in charge transport engineering for (DMA)_3_P‐derived QLEDs. Despite these efforts, (DMA)_3_P‐based QDs and their corresponding QLEDs still exhibit significantly lower efficiency, luminance, and operational lifetime compared to their (TMS)_3_P‐based counterparts. Hence, we identified the critical bottlenecks limiting the development of (DMA)_3_P‐derived QDs and corresponding QLEDs, proposing potential solutions and future research directions to overcome these limitations and advance the field.

## Author Contributions

Zhenghui Wu, Yanbing Lv, and Fei Chen conceived the idea and initiated the project. Zifeng Zhang and Jilin Deng organized the contents of InP‐based QDs. Zifeng Zhang and Fei Chen organized the contents of InP‐based QLEDs. Zifeng Zhang, Zhenghui Wu, Yanbing Lv, and Fei Chen mainly wrote this manuscript. Zifeng Zhang, Jilin Deng, Qiulei Xu, Zhenghui Wu, Yanbing Lv, Haiyang Li, and Fei Chen replied to the review's comments. Zhenghui Wu, Yanbing Lv, Baocheng Yang, Fei Chen, and Huaibin Shen contributed to the discussion and the revision of the manuscript. Zifeng Zhang, Qiulei Xu, Zhenghui Wu, Yanbing Lv, Fei Chen, and Huaibin Shen provided financial support. All authors commented on the manuscript and confirmed the submission.

## Conflicts of Interest

The authors declare no conflicts of interest.

## Data Availability

The data that support the findings of this study are available from the corresponding author upon reasonable request.
